# Current Progress of Excellent Photodetectors Based on Novel Semiconductor Nanomaterials

**DOI:** 10.3390/nano16090549

**Published:** 2026-04-30

**Authors:** Tianmeng Shang, Changxing Li, Yarong Shi, Dandan Sang, Zhanfeng Zhang, Hang Li, Qinglin Wang

**Affiliations:** 1 Key Laboratory of Quantum Materials Under Extreme Conditions in Shandong Province, School of Physics Science and Information Technology, Liaocheng University, Liaocheng 252000, China; 2 Shandong Jinhengli Mechanical Manufacturing Co., Ltd., Taian 271200, China

**Keywords:** photodetector, nanomaterial, heterojunction, detectivity, photosensitivity

## Abstract

Photodetectors have undergone widespread, gradual application. Correlation detectors with varying properties are used in diverse fields. This review systematically summarizes the principles, properties, and applications of various photoelectric detectors reported in the past five years, compares their similarities and differences, and further discusses their respective advantages and disadvantages, applicable scenarios, and development prospects. The review covers self-powered detectors, which are very convenient and widely used in consumer electronics and portable wearable devices, and discusses the structural design and photoelectric performance of devices based on P–N junctions, perovskites, silicon–polymer hybrid composites, graphene, hybrid graphene/PbS quantum dot systems, and other novel material architectures. Compound photoelectric detectors enable multifunctional integration and intellectualization. At the same time, their high sensitivity and broad-spectrum response can expand the detection wavelength range to cover the ultraviolet, visible, and infrared bands and enhance the detection of weak optical signals. Finally, this review summarizes current challenges, including cumbersome fabrication processes, susceptibility of detection stability to environmental interference, and limited functionality, and focuses on recent advances in various photodetectors, where breakthroughs are expected.

## 1. Introduction

Compared to traditional bulk counterparts, modern photodetectordevices, which convert optical signals into measurable electrical responses, exhibit tailored optoelectronic properties that enable advancements in sensitivity, spectral coverage, and energy efficiency. Photodetectors are foundational to applications spanning optical communication, environmental monitoring, biomedical imaging, and smart sensing [1]. Among emerging configurations, specialized categories such as self-powered, ultraviolet (UV), visible-light, near-infrared (NIR), P–N junction-based, broadband, solar-blind [2], quantum dot [3] and other photodetectors have gained prominence, each optimized for distinct operational demands. For self-powered photodetectors, a SnO_2_ nano-litchi-shell/n-Si heterojunction, with its superior broadband light absorption, demonstrates substantial potential for biomedical testing, environmental monitoring, and military reconnaissance applications [4]. Additionally, an ultra-thin MoO_3_/Ir/SiO_2_/Si heterojunction Schottky photodetector with self-powered functionality has been reported, showing positive photoconductivity [5]. This suggests that annealing promotes Ir diffusion into the MoO_3_ layer, increasing the probability of phonon scattering and consequently extending the range of negative photoconductivity behavior, underscoring the significance of negative photoconductive devices in optoelectronics. Self-powered photodetectors are energy-autonomous (internally converting light into electricity), highly sensitive to weak light, fast-responding (nanosecond to microsecond range), compact in structure, and stable with good environmental adaptability for long-term use [6]. Regarding UV photodetectors, scandium oxide (Sc_2_O_3_) shows great potential in high-performance solar-blind ultraviolet (SBUV) detection due to its wide bandgap and stability. Annealing is found to significantly enhance its performance, offering a new material choice for SBUV applications. Furthermore, Ga_2_O_3_-based deep-ultraviolet photoelectric synaptic memristors capable of mimicking neuromorphic functions have been realized, including a simulated 5 × 5 array, paving the way for future neuromorphic and bio-inspired computing systems [7]. UV photodetectors typically share core characteristics: high sensitivity to ultraviolet light (particularly in the UVC and UVA bands), low dark current for clear signal detection, fast response speeds (nanosecond to microsecond range) for real-time operation, excellent UV selectivity to minimize visible and infrared interference, stable performance under harsh conditions (e.g., high temperature and humidity), and compact structures suitable for miniaturization and integration. Visible-light photodetectors commonly feature high sensitivity over the 400–760 nm wavelength range, low dark current, and a high signal-to-noise ratio for accurate detection. They also offer fast response times (nanosecond to microsecond range) to meet real-time requirements, good spectral selectivity to reduce UV/IR interference, stable performance under ambient conditions, and compact designs that facilitate miniaturization and integration into electronic systems. For hybrid graphene/PbS quantum dot photodetectors, an in situ fluorination treatment using benzene carbonyl fluoride has been developed to remove bulk traps in metal chalcogenide quantum dots, thereby accelerating carrier transport. Experimental results on two types of photoelectrical conversion devices demonstrate the advantages of this treatment, offering promising prospects for the practical application of QD technology. Additionally, a methodology involving tight ionic pair formation with cationic surfactants has been introduced to shift the colloidal stabilization of metal chalcogenide complex-capped PbS QDs from long-range electrostatic to short-range steric interactions, further boosting the responsivity and detectivity of photoelectronic devices [8]. Hybrid graphene/PbS quantum dot photodetectors are characterized by the integration of graphene’s high carrier mobility with the strong light absorption of PbS quantum dots, enabling ultra-high responsivity (often >10^6^ A/W) and broadband detection from visible to near-infrared wavelengths. They exhibit fast response speeds (typically sub-microsecond), low dark current, good photostability, and a flexible, scalable structure compatible with wearable devices and integrated circuits. Some designs also enable self-powered operation, further broadening their application potential. Regarding perovskite photodetectors, halide perovskites offer great potential for broadband detection but face challenges such as ion migration and parasitic charge injection [9]. Utilizing an ultrathin ferrocenyl bis(phenyl-2-carboxylate) (FcPhc_2_) layer as a hole-blocking layer creates an energetic barrier that reduces hole injection, lowers noise spectral density, increases specific detectivity, inhibits iodine oxidation, and enhances reverse bias stability without sacrificing device response speed. Furthermore, the use of a low-toxicity antisolvent (SPA) to regulate film formation has achieved a high photoresponsivity of 1.06 A/W at 532 nm. The polarity of antisolvents significantly affects perovskite film quality and device performance, providing a useful reference for preparing high-performance perovskite photodetectors [10]. Despite advances in self-powered, perovskite, broadband, solar-blind, and hybrid graphene/PbS quantum dot photodetectors, critical challenges remain: self-powered devices deliver low output under weak light; perovskite detectors lack long-term environmental stability; broadband models struggle with uniform performance across wide spectra; solar-blind types face sensitivity-selectivity trade-offs; and hybrid graphene/PbS quantum dot devices suffer from high dark current and complex fabrication. Large-scale integration is further constrained by material compatibility across most device types. This review summarizes the latest advances in a variety of developing photodetector technologies, highlights key performance metrics, and outlines prospects for their future practical application.

## 2. Applications of Variety Photodetectors

### 2.1. Self-Powered Photodetectors

Mostly, self-powered photodetectors can function without external power. They can obtain electrical energy from light, mechanical, and other forms of energy, such as solar, pressure, and vibration. The piezoelectric–phototronic effect [11], in-turn, uses mechanical strain (e.g., from material bending) to induce a voltage that modulates the junction barrier, thereby accelerating carrier transport and improving sensitivity to weak light signals. Compared with conventional photodetectors, self-powered versions offer distinct advantages: they eliminate the need for external power, facilitating device miniaturization and portability; their energy autonomy makes them suitable for long-term, off-grid operation; and the integration of piezoelectric mechanisms can enhance performance under low illumination conditions.

**Figure 1:**
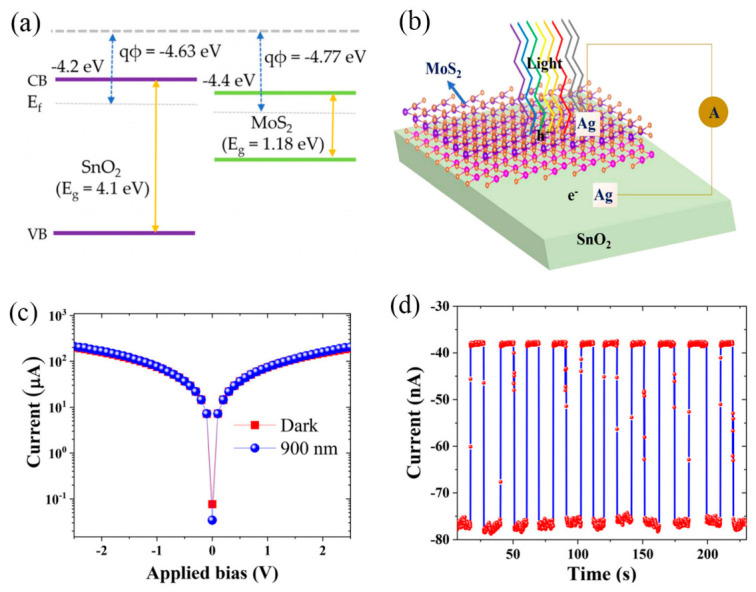
(**a**) Schematic illustration of the MoS_2_/SnO_2_ heterostructure device. The corresponding photodetection mechanism is explained by the energy band diagram shown in (**b**). A distinct abrupt variation in photocurrent at zero bias (**c**) verifies that the device can operate in a self-powered mode driven by the built-in voltage at the heterojunction interface. Furthermore, the device’s stability and reproducibility were evaluated through multiple on–off cycles under modulated illumination. As seen in (**d**), the consistent photocurrent response across repeated cycles confirms the photodetector’s stability for practical device applications [15]. Copyright 2022, AIP Publishing.

Hu et al. fabricated a self-powered suspended Pd-reduced graphene oxide-Ti (Pd-rGO-Ti) photodetector. They suggested that further reductions in channel width and atmospheric pressure could improve device performance. Benefiting from facile preparation, low manufacturing cost, scalability for mass production and an ultra-broadband photoresponse, such Pd-rGO-Ti photodetectors possess extensive application prospects in high-performance integrated optoelectronic systems [12]. Ling and co-workers fabricated a SnO_2_ nano-litchi-shell/n-Si heterojunction with boosted broadband light absorption and applied it to self-powered photodetection. Furthermore, they assessed the device’s performance in near-infrared low-signal imaging and fluorescence detection, conclu that this heterojunction photodetector exhibits remarkable potential for biomedical detection, environmental monitoring and military reconnaissance applications [4]. Jing et al. demonstrated that a ZnO nanocrystal (NC) array can spontaneously generate carriers and successfully achieve detection at zero bias under UV irradiation. In their work, the structure was fabricated on two different substrates—silicon (Si) and GaN—to validate its performance [13]. Pius Augustine et al. fabricated an MoS_2_/SnO_2_ heterostructure device. Figure 1a shows the energy band structures of MoS_2_ and SnO_2_ before the formation of the heterojunction, where the conduction band edges are determined as −4.4 eV and −4.2 eV, respectively. The MoS_2_/SnO_2_ heterostructure promotes selective carrier transport by oxidation of a Sn film in ambient doping electrons into the CB of the SnO_2_ film while also significantly localizing holes in the VB of the heterostructure. Thus, strongly enhanced n-type transport was achieved, which is suitable for photodetector and electron-transporting layer applications in photovoltaic devices [14]. The photodetection mechanism of the device is illustrated in Figure 1b. Under illumination with light of varying wavelengths, photons with energy exceeding the bandgap of MoS_2_ produce electron-hole pairs, which are separated and transported to the electrodes via the built-in electric field at the MoS_2_/SnO_2_ heterointerface. As shown in Figure 1c, the current-voltage (I-V) characteristics of the MoS_2_/SnO_2_ heterostructure device were characterized under dark conditions and 900 nm light irradiation. To evaluate the device’s stability and reproducibility, on–off cycling tests were performed over multiple illumination cycles. The repeatable photocurrent response observed in Figure 1d confirms the device’s stability for practical applications.

Mohamed A. et al. prepared an ultrathin self-powered Schottky photodetector based on a MoO_3_/Ir/SiO_2_/Si heterojunction, demonstrating its positive photoconductivity performance. The elevated phonon-scattering probability and the resulting prolonged negative photoconductivity response underscore the notable significance of negative photoconductive devices in optoelectronics [16].

**Figure 2:**
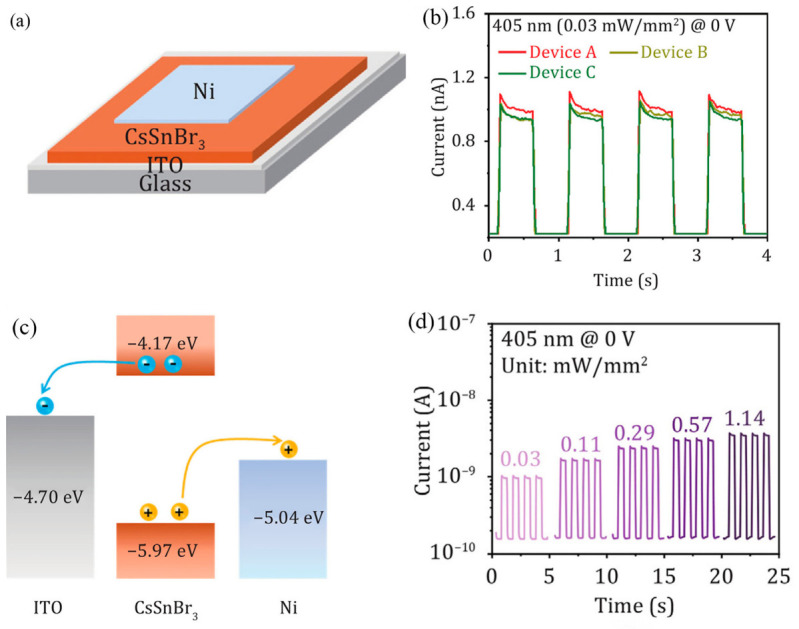
Self-powered photodetection behaviors of the CsSnBr_3_/ITO heterostructure film: (**a**) schematic illustration of the CsSnBr_3_/ITO heterostructure-based photodetector; (**b**) self-powered photoresponse characteristics of the three fabricated devices; (**c**) energy band diagrams of the self-powered photodetector with a Ni/CsSnBr_3_/ITO structure; (**d**) current–time (I-t) profiles of the CsSnBr_3_/ITO heterostructure photodetector (CsSnBr_3_ film grown for 9 min) under 405 nm laser irradiation with varying intensities [19]. Copyright 2023, Elsevier.

Pradip et al. noted that while self-powered photodetectors have attracted widespread attention for their broad applications, their fabrication is often complex and costly. They introduced a new photoelectrochemical platform based on two-dimensional bismuth oxide selenide (Bi_2_O_2_Se), which exhibits high performance metrics, including high responsivity, broad spectrum sensitivity, fast response, and good stability, positioning it as a promising candidate for future high-efficiency optoelectronic devices [17]. Hu et al. detailed the fabrication of self-powered photodetectors via a one-step spin-coating process for organic bulk heterojunction films. The study explored related mechanisms and demonstrated the device’s high performance, providing both theoretical and experimental support for advancing self-powered photodetectors in the field of organic materials [18]. Liu et al. designed and demonstrated a high-performance omnidirectional self-powered photodetector based on a CsSnBr_3_/indium tin oxide (ITO) heterostructure film. To study the photodetection performance of the CsSnBr_3_/ITO heterostructure, three photodetectors with a vertical Ni/CsSnBr_3_/ITO structure were fabricated, as shown in Figure 2a. The self-powered photoresponse of the three devices is presented in Figure 2b. Figure 2c shows the energy band diagrams of the ITO/CsSnBr_3_/Ni-based photodetector. The appropriate band alignment among ITO, CsSnBr_3_, and Ni results in the formation of a built-in electric field at the interface, facilitating the separation of photogenerated carriers. Additionally, with the increase in the growth time of the CsSnBr_3_ film, the grain size increases, the GBs density decreases, the density of the localized states in which act as trap states can be beneficially tuned and the recombination at the grain boundary is effectively inhibited, which contributes to the improvement of photodetection performance [14]. As shown in Figure 2d, the photocurrent increases monotonically with the intensity of the 405 nm laser, indicating the good stability and reproducibility of the fabricated CsSnBr_3_/ITO heterostructure photodetector. It was also observed that lower grain boundary (GB) density leads to a larger photocurrent; for example, Device A, which has almost no GBs, exhibits the highest photocurrent. The fabricated photodetector further demonstrated excellent omnidirectional self-powered photodetection performance. Overall, the results confirm that this work provides an effective approach to realizing high-performance, self-powered omnidirectional photodetectors.

Self-powered photodetectors eliminate the need for external power supplies, making them ideal for battery-free ITO nodes, wearable electronics, and remote sensing. Unlike conventional bias-driven devices, SPPs operate via photovoltaic, pyroelectric, or triboelectric–photonic coupling effects, with emerging lead-free perovskite and 2D van der Waals heterostructures delivering record detectivities. However, most demonstrations suffer from low photocurrent density, narrow spectral response, and poor long-term stability under ambient conditions. Integrating SPPs with on-chip signal processing and multimodal sensing remains the most promising path for practical deployment.

### 2.2. UV Photodetector

A UV photodetector is an optoelectronic device that converts ultraviolet radiation (wavelength range: 10–400 nm) into measurable electrical signals, serving as a critical component for UV sensing, monitoring, and detection in scientific and industrial applications [20]. Its operating principle is based on the photoelectric effect and semiconductor photoconductivity, with type-II band alignment at the heterojunction interface of wide-bandgap semiconductors (e.g., Ga_2_O_3_/4H-SiC), further facilitating ultrafast separation of photogenerated electron-hole pairs and suppressing carrier recombination.

Liu et al. successfully fabricated composite nanomaterials comprising TiO_2_ nanotube arrays (TiO_2_ NTAs) and polyaniline (PANI) on titanium substrates via anodic oxidation. The results demonstrated that the TiO_2_ NTA/PANI composites exhibit excellent UV photosensitivity, response, stability, and reproducibility. This work provided a theoretical basis for the application of metal oxides in UV photodetectors, contributing significantly to their development [21]. Yannick et al. reported the realization of real-time ultraviolet photodetectors based on metal-semiconductor-metal (MSM) configurations. The fabricated photosensor exhibits high responsivity (R), low response time (t_RES_), and a favorable noise equivalent power (NEP) value [22]. Nanostructured pure and molybdenum (Mo)-doped nickel oxide (NiO) thin films with varying Mo concentrations were successfully deposited onto ITO substrates for use in UV photodetectors [23].

Rong-Ming Ko et al. developed a novel strategy to alleviate the trade-off between dark current and photocurrent in indium gallium zinc oxide (IGZO) thin-film transistors (TFTs), which are widely applied in ultraviolet photodetectors and other optoelectronic devices, via the introduction of a stacked Pt/NiO double capping layer (CL) [24]. Han et al. demonstrated gate-modulated p-GaN/AlGaN/GaN ultraviolet photodetectors (UVPDs) with ultrahigh responsivity on Si substrates, which integrated a high-transmittance ITO gate electrode. The two-dimensional electron gas (2DEG) confined in the quantum well of the polarized AlGaN/GaN heterojunction was effectively depleted under the modulation of the p-GaN gate, leading to an ultrahigh photo-to-dark current ratio (PDCR) of 3.2 × 10^5^. By integrating the high-transmittance ITO gate with the mature commercial structure of p-GaN/AlGaN/GaN high-electron-mobility transistors (HEMTs), this device design provides a feasible and promising strategy for the mass fabrication of low-cost, high-performance UVPDs in the future [25]. Cen et al. highlighted the importance of the hole transport layer (HTL) in enhancing the performance of ultraviolet photodetectors (UV PDs). In their study, a graphene oxide (GO)-modified CuI p-type layer was employed as a hole extractor for UV PDs based on ZnO nanorod arrays. The fabricated FTO/ZnO NRs/CuI@GO/Au device demonstrated excellent performance. The addition of GO optimizes the work function and band structure of CuI and suppresses the pyroelectric effect, providing a new strategy for optimizing CuI as an HTL [26]

The effect of W doping on the performance of ZnO/Si-based photodetectors was investigated. It was found that a W dopant concentration of 2 at% is optimal for a high-performance ZnO:W/Si heterojunction photodetector, as it facilitates suppressed recombination and enhanced carrier separation within the depletion region [27]. Manh Hoang Tran et al. noted that zirconium-oxo clusters (ZOCs), characterized by high stability and low trap density, have not yet been utilized for UV detection. Their work proposes the application of self-assembled ZOC square flakes in deep-UV photodetectors. The ZOC@PEI composite, integrated into a MSM device, demonstrates excellent UV photoresponse, indicating the potential of ZOCs for sensitive, low-power UV photodetection [28]. K. Ozel and A. Yildiz fabricated transparent photodetectors (PDs) using undoped zinc oxide (ZO) and zinc oxide doped with 3 atomic percent (at.%) aluminum (AZO) thin films. Both ZO and AZO films were deposited on fluorine-doped tin oxide (FTO) substrates via spin-coating to explore how Al doping affects the UV detection performance of the photodetector devices [29]. Liang et al. proposed a strategy to improve the performance of Lu_2_O_3_-based DUV PDs, the Ti_3_C_2_T_x_/Lu_2_O_3_/GaN DUV photodetector exhibited excellent performance at 0 V bias. This work proposes new ideas for the application of wide-bandgap Lu_2_O_3_ semiconductors in high-performance DUV photodetectors [30]. Li et al. fabricated SBUV PDs with b-Ga_2_O_3_ films grown via metal–organic chemical vapor deposition (MOCVD) and demonstrated the significant role of non-VO factors in shaping the time-resolved detection performance of these devices [31].

**Figure 3:**
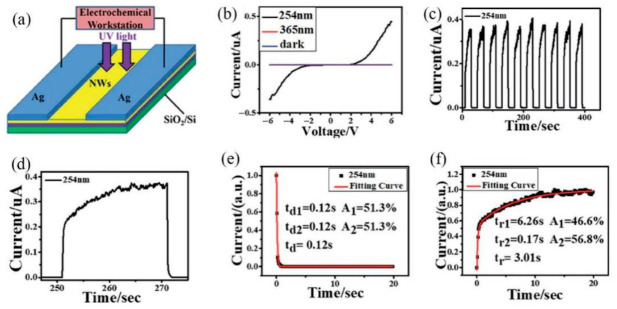
Device schematic and photoresponse characterization. (**a**) Schematic diagram of the β-Ga_2_O_3_ UV photodetector; (**b**) I-V characteristics of the β-Ga_2_O_3_ nanowire network photodetector in the dark and under 365 nm and 254 nm light irradiation, respectively; (**c**) I-T characteristics of the β-Ga_2_O_3_ nanowire network photodetector under 6 V bias with 254 nm light irradiation; (**d**–**f**) Relationship between the rise/decay time and normalized current of the β-Ga_2_O_3_ nanowire network photodetector [38]. Copyright 2021, Royal Society of Chemistry.

Wang et al. studied the UV detection and gas-sensing behaviors of hydrothermally grown ZnO nanorod arrays (ZNAs) on silver nanowire meshes (AgNMs), focusing on the influence of zinc acetate precursor concentration. Further in-depth experiments were performed, and these findings underscore the feasibility of ZNA-AgNM for high-performance UV photodetectors and hydrogen sensors, offering a new route to advanced sensing devices with enhanced performance and multifunctionality [32]. Wu et al. utilized high-Al-content β-(Al_0.4_Ga_0.6_)_2_O_3_/Al_0.32_Ga_0.68_N films, prepared via the thermal oxidation of AlGaN layers, to fabricate UVC/UVB dual-band photodetectors. The devices exhibited a notable pyroelectric effect and achieved high sensitivity, combined with a fast response time, in UV photodetection [33]. Zhang et al. highlighted the significant promise of gallium oxide (Ga_2_O_3_)-based solar-blind photodetectors (SBPDs). Through the preparation of high-quality single-crystalline β-Ga_2_O_3_ films, they constructed a self-powered n-Si/n-Ga_2_O_3_/p-Li:NiO dual-heterojunction device. This detector exhibited outstanding responsivity and durable stability, with a well-elucidated operating principle: the superimposed dual built-in electric fields effectively promote the separation and migration of photogenerated charge carriers. The strategy presents a viable route for advancing high-performance Ga_2_O_3_-based photovoltaic detection systems [34]. Shinji et al. fabricated an n-p-n structure based on a β-Ga_2_O_3_/NiO/β-Ga_2_O_3_ junction. Ga_2_O_3_ has recently attracted attention as a next-generation power semiconductor material. Among its polymorphs, β-Ga_2_O_3_ possesses a bandgap of 4.9 eV and is the most thermodynamically stable; it has been widely studied for power devices and ultraviolet photodetectors due to these favorable physical properties [35]. Jiang et al. indicated that Sc_2_O_3_ exhibits considerable potential for high-performance SBUV photodetectors owing to its wide bandgap and excellent stability. They tuned the bandgap to 4.65 eV using a ternary-alloy film method and fabricated a high-performance self-powered photodetector with an annealed Sc_0.74_In_1.26_O_3_ photosensitive layer. It was found that annealing significantly enhances device performance, providing a new material option for SBUV photodetectors [36]. Liang et al. noted that while deep ultraviolet (DUV) radiation holds broad application potential, conventional DUV photodetectors are often limited by cost and response speed. Their paper presents Ga_2_O_3_-based DUV photoelectric synaptic memristors that mimic neuromorphic functions. A fabricated 5 × 5 array demonstrates the feasibility of this approach, paving the way for future neuromorphic and bio-inspired computing systems [7]. Huang et al. highlighted that Ga_2_O_3_ is a promising wide-bandgap semiconductor for next-generation applications, such as solar-blind UV detection. However, its development has been hindered by a lack of viable isovalent-substitution doping strategies. The study showed that trivalent lanthanide doping in Ga_2_O_3_ can regulate the bandgap, and an ITO/Ga_2_O_3_:Ln/Au photodetector demonstrated significant optoelectronic potential, exhibiting low dark current and a fast response [37]. Wu et al. identified monoclinic β-Ga_2_O_3_ as a promising ultra-wide bandgap semiconductor for deep ultraviolet detection. In their work, self-catalyzed lateral nanowire networks of β-Ga_2_O_3_ were rapidly synthesized on an insulating substrate via a simple chemical vapor deposition (CVD) method. MSM photodetectors based on these networks demonstrate fast response times, indicating potential for next-generation semiconductor nanoelectronics. A typical MSM photodetector with an approximate 2 mm gap between two Ag electrodes is shown in Figure 3a. Figure 3b presents the I-V characteristics of the device under dark conditions and under 254 nm and 365 nm illumination at a bias of 6 V. It is evident that the current under 254 nm illumination is significantly higher than both the dark current and the current under 365 nm illumination, indicating that 254 nm light substantially enhances the conductivity of the nanowire network device. Figure 3c,d display the I-T characteristics of the photodetector under a 6 V bias. The responsivity (Rλ) is defined as the ratio of the photocurrent to the incident optical power on the photodetector, expressed as Rλ = (Iph − Idark)/(P0·A). Finally, Figure 3e,f show the photoresponse behavior of the β-Ga_2_O_3_ nanowire network photodetector.

**Figure 4:**
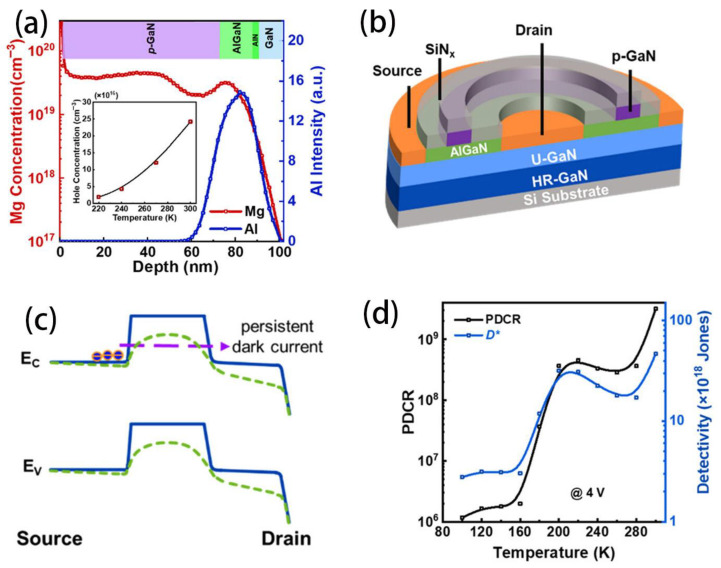
(**a**) Secondary ion mass spectrometry depth profile of the p-GaN/AlGaN/GaN heterostructure. Inset: Hole concentration in the p-GaN layer as a function of temperature. (**b**) 3D cross-sectional view of the device schematic of a p-GaN HEMT photodetector. (**c**) Schematic energy band diagram along the lateral channel direction after switching off UV illumination. Solid and dashed lines represent the cases at room temperature and low temperature, respectively. (**d**) PDCR and D as a function of temperature under a bias of 4 V [40]. Copyright 2025, AIP Publishing.

Wang et al. investigated the photoresponsivity of a high-performance p-GaN high-electron mobility transistor (HEMT)-based ultraviolet photodetector at low temperatures. As illustrated in the inset of Figure 4a, the average bulk hole concentration in the p-GaN gate decreases significantly as the temperature drops from 300 K to 220 K, due to the freeze-out of Mg dopants. As a result, the potential of the two-dimensional electron gas (2DEG) beneath the p-GaN gate rises, lowering the barrier in the depletion region and leading to a large dark current. Absorption of light by p-GaN increases when its surface area increases. Both the surface area and the band gap can be increased when the particle size is reduced to nanoscale dimensions. Therefore, attention has been paid to prevent the agglomeration of p-GaN particles in order to improve their optical properties [39]. Figure 4b shows the schematic three-dimensional structure of the p-GaN HEMT photodetector. The mechanism of persistent photoconductivity (PPC) behavior in the p-GaN HEMT photodetector is explained using the energy band diagrams presented in Figure 4c. Figure 4d plots the PDCR and detectivity (D) as a function of temperature. The device demonstrated an extraordinary responsivity of 10^6^ A/W at 100 K, highlighting its strong potential for operation in frigid environments. This finding provides constructive insight into the persistent photoconductivity behavior and can contribute to further improvements in device performance.

Recent UV photodetector research has pivoted from bulk material discovery to interface engineering and novel architectures. While ultra-wide bandgap semiconductors (Ga_2_O_3_, AlGaN) offer intrinsic solar-blindness, their integration is hindered by p-type doping bottlenecks and lattice mismatches. Conversely, 2D materials and self-powered designs offer flexibility but suffer from a persistent trade-off between sensitivity and speed due to weak internal fields. Critical advances now center on hybrid heterojunctions, e.g., ZnO/Bi_2_O_3_ or polymer/Ga_2_O_3_, to enhance carrier separation and bypass inorganic incompatibilities. Additionally, photoelectrochemical (PEC) devices offer a disruptive paradigm for ultra-low dark current, though integration lags behind that of solid-state systems. The field is thus defined by a strategic divergence: optimizing interfacial dynamics over intrinsic material limits.

### 2.3. Visible-Light Photodetector

A visible-light photodetector is an optoelectronic device that converts visible light (wavelength range: 400–760 nm) into measurable electrical signals, serving as a core component for light sensing and imaging [41]. When visible-light photons strike the detector’s active material, they excite electrons from the valence band to the conduction band, generating electron–hole pairs. For high-sensitivity designs, nanostructured materials such as graphene composites [42] can enhance light–matter interactions and improve carrier-generation efficiency. Compared with other types of photodetectors, visible-light photodetectors offer distinct advantages. They match the spectral response of the human eye, making them suitable for visual imaging applications. They have extensive applications in various fields. In industrial settings, they enable machine vision systems for product sorting and defect detection on production lines and support semiconductor manufacturing by monitoring photoresist UV-curing processes. In healthcare, they are integrated into medical imaging devices, such as endoscopes for tissue visualization, to verify the effectiveness of UV sterilization in hospitals and to enable precise UV dose monitoring during phototherapy. In aerospace and automotive electronics, they assist in advanced driver-assistance systems (ADAS) —including lane departure warning and pedestrian detection—and support visible-light communication (VLC) [43] for high-speed indoor data transmission using LED sources. In specialized fields, they contribute to precision agriculture by monitoring crop chlorophyll content through visible-light reflectance, aiding environmental protection by detecting pollutants such as heavy metal ions in water quality monitoring, enhancing security surveillance with high-resolution, low-light cameras, enabling non-destructive inspection of artworks and manuscripts for cultural heritage preservation, and supporting remote or portable sensing applications through self-powered designs.

**Figure 5:**
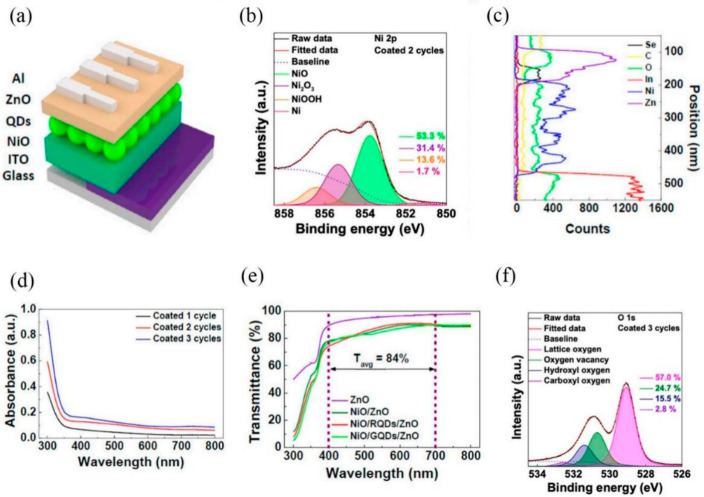
(**a**) Schematic structure of the fabricated photodiode. (**b**) XPS spectra of NiO layers deposited with varying numbers of coating cycles. (**c**) EDS line-scan profile of the photodiode cross-section. (**d**) Absorption spectra of the NiO layers. (**e**) Transmittance spectra of the corresponding device layers; Ni_2_P_3/2_ and O 1s core-level XPS spectra are shown in the insets. (**f**) O 1s core-level spectra [45]. Copyright 2024, MDPI.

Po-Hsien Tseng et al. fabricated a high-sensitivity visible-light photodetector using a thin-film structure composed of low-cost AZO and n-type silicon. The thickness of the AZO layer can be accurately regulated by interrupted-flow atomic layer deposition (ALD), enabling spectral response matching to the target wavelengths in the visible region. Given its notable photoelectric conversion efficiency and straightforward thin-film design, this device holds promise for future applications in light-intensity measurement, such as colorimetry or fluorometry [44]. Seong Jae Kang et al. highlighted that visible-light photodetectors incorporating transparent metal oxide layers have been extensively studied. Figure 5a illustrates a schematic of the photodiodes fabricated in this work. Figure 5b displays the Ni_2_P_3/2_ peaks, which are primarily composed of Ni^2+^ and Ni^3+^ components. The TEM and EDS data presented in Figure 5c confirm the specified layer thicknesses and indicate no interlayer interference. This study aimed to improve the visible-light detection capability of NiO/ZnO photodiodes by inserting a quantum dot (QD) layer. The incorporated QDs enabled wavelength-selective light detection. Furthermore, XPS and UPS analyses were employed to investigate the origin of the photocurrent, validating QDs as an effective strategy for visible-light detection in transparent optoelectronic devices. Figure 5d shows that while NiO and ZnO absorb mainly in the UV region, red quantum dots (RQDs) and green quantum dots (GQDs) absorb light within the targeted visible range. Figure 5e presents the transmittance data of the devices, which decreased with successive layer stacking. Finally, Figure 5f displays the O 1s peaks of NiO, deconvoluted into lattice oxygen (OL), oxygen vacancy (OV), hydroxyl oxygen (OH), and carboxyl oxygen (OC) components.

CuGaSe_2_ is a promising material for solar cell applications; notably, the bandgap of two-dimensional hexagonal CuBi_x_Ga_1-x_Se_2_ nanosheets can be tuned by substituting Ga with Bi. Variations in the Bi/Ga ratio induce structural changes, and the resulting nanosheets exhibit broad absorption, reduced optical absorption with increasing Bi content, and an enhanced photocurrent response under white-light illumination. These properties are crucial for optoelectronic devices, particularly photodetectors [45]. Prabhukrupa et al. noted that two functional materials, Ag_2_S and In_2_Se_3_, have attracted significant research attention. A visible-light photodetector based on Ag_2_S/In_2_Se_3_ heterostructure films was fabricated at room temperature. Following the annealing of the bilayer film at various temperatures, the sample annealed at 250 °C demonstrated improved photoconductivity, along with enhanced hydrophilicity and crystallinity, rendering it suitable for visible-light photodetection [46].

Visible-light photodetectors are core components for imaging, biosensing and optical communications, outperforming conventional silicon-based devices in low-light sensitivity and spectral tunability. While early 2D VLPDs suffered from poor interface engineering and uncontrollable material growth, recent breakthroughs have unlocked unprecedented performance via rational heterostructure design and precise growth control. Notably, ferroelectric CIPS/MoS_2_ heterojunctions achieve a record responsivity of 730 A·W^−1^ and a photocurrent-to-dark current ratio >10^4^; controllably grown SbI_3_ and single-crystalline Sb_2_Te_3_ nanosheets deliver excellent detectivity up to 8.4 × 10^10^ Jones. Most importantly, self-powered 2D perovskite/InSe waveguide PDs realize monolithic integration with SiN photonic circuits, enabling label-free DNA detection at zero bias.

### 2.4. Near-Infrared Photodetectors

A NIR photodetector is an optoelectronic device that converts near-infrared light (wavelength range: 700–2500 nm) into measurable electrical signals, such as current or voltage. Near-infrared photodetectors include different types. Common types are detectors based on organic semiconductors, organic materials, or novel materials. Near-infrared organic photodetectors (NIR OPDs) exhibit distinctive advantages including, tunable optoelectronic performance, facile processing, flexible substrate compatibility, and room-temperature operation. Thus, they are promising candidates for future electronics, driven by the rising demand for wearable devices and biomedical applications [47]. Near-infrared photodetectors play a crucial role in fields such as optical fiber communications and biomedical applications [48]. In optical fiber communication systems, these devices serve a critical function by converting near-infrared optical signals into corresponding electrical impulses, thereby supporting high-speed data transmission and advancing the development of 5G and 6G networks. In biomedical fields, they enable non-invasive diagnostic techniques, such as near-infrared spectroscopy, which can be applied to track blood oxygen levels, identify tumor tissues, and examine biological structures. NIR OPDs offer a range of unique advantages, including customizable photoresponse properties, simple manufacturing processes, compatibility with bendable substrates, and stable operation under ambient conditions [47]. Given the growing demand for wearable electronics and biomedical devices, NIR OPDs are promising candidates for next-generation electronic products.

**Figure 6:**
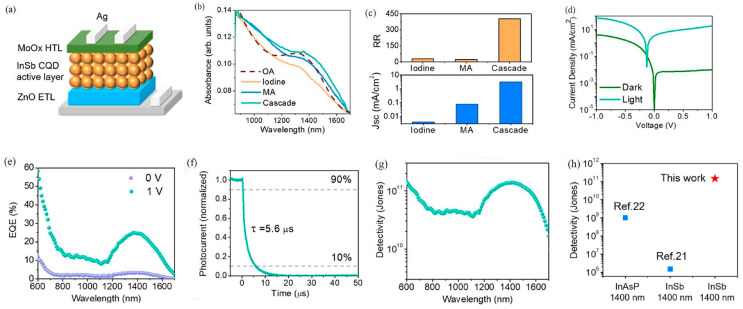
(**a**) Device schematic; (**b**) absorption spectra of OA-capped and ligand-exchanged CQD films. (**c**) Rectification ratio and short-circuit current comparison of photodetectors with different surface-modified CQDs as the active layer. (**d**) Current density-voltage curves and (**e**) EQE spectra of cascade-exchanged InSb CQD photodetectors under 0 V and 1 V reverse biases. (**f**) Transient photocurrent response of InSb CQD photodetectors (0.2 mm^2^ pixel area) at 1 V bias. (**g**) Specific detectivity versus wavelength. (**h**) Specific detectivity comparison between this work and reported III-V CQD photodetectors at 1400 nm [50]. Copyright 2024, John Wiley and Sons.

Zhong et al. fabricated a high-performance ZnO/PbS heterojunction photodetector by spin-coating PbS colloidal quantum dots (CQDs) onto the surface of a vertically aligned ZnO nanowire (NW) array grown hydrothermally on an ITO substrate. The excellent performance and low cost of this nanocomposite-based photodetector indicate its potential for broad applications, ranging from medical diagnosis to environmental monitoring [49]. Zhang et al. noted that heavy-metal-free III-V colloidal CQDs are promising candidates for solution-processed short-wave infrared (SWIR) photodetectors. By employing solvent engineering to obtain uniform InSb CQDs with a 0.89 eV bandgap, along with a surface reconstruction strategy to reduce trap density and enhance electronic coupling, the resulting photodetectors achieved an external quantum efficiency of 25% at 1400 nm-the highest reported among III-V CQD photodetectors in this spectral region. Photodetectors featuring an n-i-p configuration (Figure 6a) were constructed with InSb colloidal CQDs serving as the active layer, with ZnO and MoO_x_ employed as the electron transport layer (ETL) and HTL, respectively. In comparison to oleic acid-capped CQD films, the absorption peaks of methylamine-treated and cascade-exchanged CQD films exhibited a slight red shift (Figure 6b), which further confirms the strengthened coupling between CQDs. Figure 6c presents a comparison of the current-voltage (I-V) properties of devices fabricated using CQDs with varied surface modification strategies. The performance of photodetectors utilizing cascade-exchanged InSb CQDs as the active layer is depicted in Figure 6d–g. Notably, the detectivity of these InSb CQD photodetectors was enhanced by five orders of magnitude relative to previously reported InSb-based devices, and it exceeded that of InAsP photodetectors within the same spectral range by 140 times (Figure 6h). The operational stability of the InSb CQD photodetectors was assessed by tracking the photocurrent and dark current under a reverse bias of 0.1 V.

Hossein Jeddi et al. conducted both experimental and theoretical investigations to explore the long-wavelength infrared (LWIR) photoresponse of photodetectors based on arrays consisting of three million InP nanowires, in which InAsP quantum disks were axially embedded [51]. Yan et al. presented an inverse design approach to enhance the performance of multi-diameter InAs nanowire array ultra-broadband photodetectors via localized surface plasmon resonances. By optimizing nanowire diameters, high absorption was achieved across a wide wavelength range. The introduction of indium tin oxide nanoparticles further improved absorption and reduced dark current density. This work may pave the way for the development of ultra-broadband, high-responsivity infrared photodetectors [52]. Zach D. Merino et al. emphasized that rapid, accurate NIR light detection is critical in modern society and that various platforms are being explored to enhance NIR photodetection performance. While challenges remain in current approaches, their work investigates the potential of tailored semiconductor nanocrystals (NCs) in planar metal-semiconductor-metal (MSM) photodetectors, presenting a protocol for synthesizing and testing non-stoichiometric tungsten oxide nanorods and cesium-doped tungsten oxide hexagonal nanoprisms [53]. Vuong et al. noted that infrared photodetectors are vital in a wide range of practical applications. Their study developed a high-performance yet low-cost IR photodetector using up-conversion microparticles (UCPMs) and reduced graphene oxide (RGO). Testing confirmed the device’s sensitivity to IR light and its good stability under ambient conditions, demonstrating that this combination enhances the photodetector’s light-absorbing capability [54].

**Figure 7:**
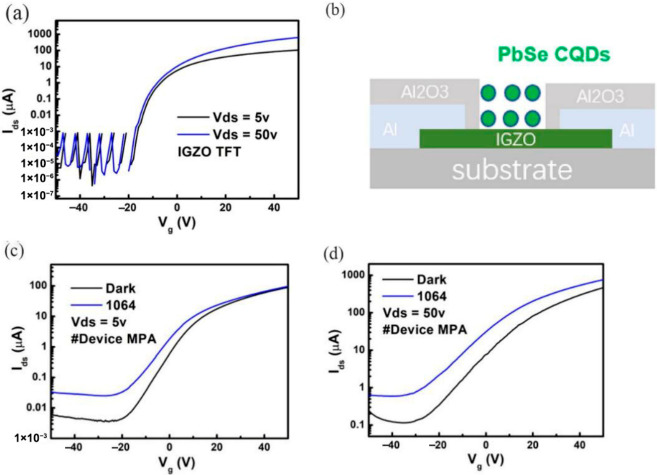
(**a**) Transfer curve of a pure IGZO thin-film transistor. (**b**) Schematic of the IGZO-PbSe quantum dot infrared photodetector. (**c**) Transfer curve of the MPA-exchanged device under 1064 nm laser illumination (34.0 mW/cm^2^). (**d**) Transfer curve of the MPA-exchanged device at $V_{ds}=50$ V under 1064 nm laser illumination (34.0 mW/cm^2^) [56]. Infrared (IR) sensors are widely used for detecting infrared radiation; however, current third-generation IR detector technology still faces significant technical challenges. Infrared radiation is divided into different transmission windows, and IR detectors are employed in diverse applications. At present, high-performance IR imaging primarily relies on specific epitaxially grown structures, whereas nanostructures and nanomaterials offer promising pathways to improve detector performance. This article provides an overview of IR sensors, including their types, materials, and applications, and concludes with a summary and outlook [57]. Copyright 2025, MDPI.

Lead-free III-V colloidal CQDs are promising for near-infrared to short-wave infrared (NIR-SWIR) photodetection, but they encounter surface-passivation challenges, especially for larger-sized CQDs. A mixed-halide passivation strategy has been developed for large InAs CQDs; this doubles their anti-oxidation ability, achieves a hole mobility of 0.03 cm^2^V^−1^s^−1^, improves operating stability, and results in photodetectors exhibiting an external quantum efficiency (EQE) of 75% and a response time of 10 ns at 1140 nm [55]. Zheng et al. fabricated an infrared photodetector based on an IGZO-PbSe CQDs heterojunction, in which the PbSe CQDs were capped with ligands MPA, TBAI, or EDT. The photodetection performance of devices with different ligand treatments was studied and compared to identify the optimal surface ligand for PbSe CQD-based photodetection. To establish the device framework, a thin-film transistor (TFT) with a pure IGZO thin film was first prepared; its performance confirmed that the IGZO layer functions effectively as a conduction channel, as shown in Figure 7a. Subsequently, the IGZO semiconductor layer was used as the conduction layer to construct an IGZO-PbSe heterojunction, the schematic of which is presented in Figure 7b. Figure 7c shows the transfer curve of the MPA-exchanged device under 1064 nm laser illumination (34.0 mW/cm^2^) at Vds = 5 V. Figure 7d displays the transfer curves of MPA-exchanged devices under illumination at Vds = 50 V, respectively. This work provides useful, solid insights that can advance the optoelectronic application of PbSe CQDs.

Recent NIR photodetector research reveals a clear trade-off between speed and sensitivity across material platforms. Bi_2_Se_3_/n-Si Schottky junctions excel in telecom-speed response (126 ns) due to efficient thermionic emission, yet they suffer from low responsivity (~48 mA/W) owing to rapid carrier relaxation inherent to narrow-bandgap topological insulators. In contrast, for PbS CQD devices utilizing EMIMSCN ionic passivation, superior detectivity and lower dark current are achievable, though concerns regarding Pb-toxicity and operational stability persist. On the flexibility front, while plasmonic-enhanced OPDs extend detection beyond 1000 nm, the added fabrication complexity undermines their cost advantage. A promising emerging solution lies in multimodal back-to-back organic diodes, which offer voltage-tunable VIS/NIR switching within a single pixel, effectively decoupling spectral adaptation from material rigidity.

### 2.5. P-N Photodetectors

A P-N photodetector is a basic optoelectronic device that operates on the principle of a P-N junction. It is fabricated by doping a semiconductor material to form a p-type region with an excess of holes and an n-type region with an excess of electrons. It converts incident light-spanning ultraviolet to infrared wavelengths-into measurable electrical signals, such as current or voltage, by exploiting the photoelectric effect at the junction. To improve sensitivity, efforts are often directed toward refining the P-N junction architecture, advancing device design, and boosting signal processing performance. Strategies may include using materials with high absorption coefficients, forming heterojunctions, and minimizing junction dimensions. Thanks to their stable operation, economic efficiency, and seamless integration with electronic circuits, P-N junction photodetectors are indispensable and extensively employed in a variety of applications [58]. In biomedical engineering, they can facilitate non-invasive diagnostics, such as blood oxygen monitoring in pulse oximeters and biological sample analysis in optical biosensors. They are also employed in environmental monitoring devices-including gas sensors and UV detectors [59], for air quality assessment, hazardous gas detection, and ultraviolet radiation measurement, as well as in scientific research instruments for optical spectroscopy and light intensity quantification. P-N photodetector can also can be combined with other photodetectors and applied in different fields. The all-oxide P-N junction composed of NiO and Ga_2_O_3_ demonstrates favorable stability and reproducibility under air, oxygen, and vacuum conditions. Such findings provide an economical and appropriate route to scale up the manufacturing of self-powered UV photodetectors.

**Figure 8:**
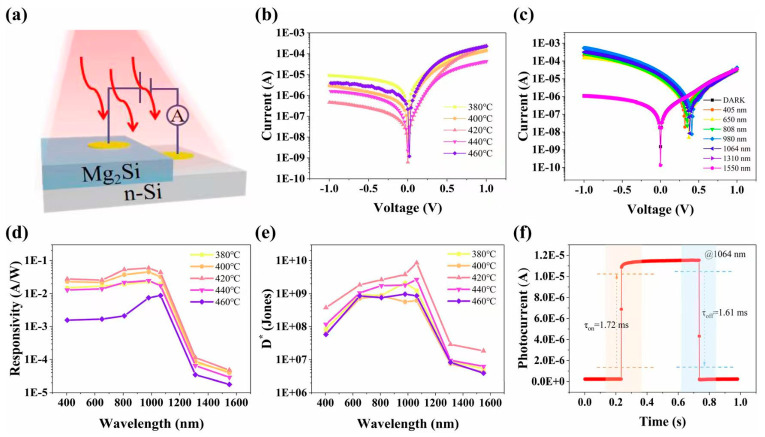
Photoelectric performance of Mg_2_Si/Si heterojunction photodetectors. (**a**) Device structure schematic. (**b**) Comparison of dark currents for devices subjected to annealing at various temperatures. (**c**) Photocurrent and dark current test results of the device (annealed at 420 °C for 8 h) under illumination of different wavelengths. (**d**,**e**) Responsivity (**d**) and specific detectivity (**e**) of devices prepared at different annealing temperatures as a function of wavelength. (**f**) Response time of the device measured at 1064 nm [63]. Copyright 2024, Royal Society of Chemistry.

Niemcharoen et al. investigated the microstructure of ITO by analyzing its physical properties across different deposition durations. The results indicate that ITO thin films exhibit approximately 97% transparency when grown for 1 h and annealed at 500 °C. XRD patterns for the sample sputtered for 60 min show characteristic ITO peaks corresponding to the (222), (400), (622), and (441) crystal planes. The objective of this study was to examine the physical properties of ITO films deposited via RF sputtering under varying sputtering times [60]. In the current work, Khawla et al. fabricated an NiO NPs/Si heterojunction photodetector by drop-casting NiO nanomaterials synthesized via laser ablation in water onto a silicon substrate. The effect of laser energy on the preparation of NiO nanoparticles was systematically investigated. This technique enables the production of nanoparticles by ablating metals or metal oxides in water and other solvents, yielding stable colloidal solutions of nan-sized materials without the use of surfactants. Compared with other methods, this approach offers multiple advantages, including simplicity, low cost, high precision, and efficiency [61]. Chao et al. presented a high-performance broadband perovskite photodetector (PPD) prepared by solution processing. The device uses MO nanoparticles as a hole-blocking layer (HBL) on the ITO electrode, and PC_61_ BM serves as an additional HBL on the metal electrode side to effectively reduce dark current. The results show that a TiO_2_ NP layer is suitable for visible-to-near-infrared (vis-NIR) photodetection, while an SnO_2_ NP layer performs well for UV-vis-NIR photodetectors. This facile solution-processed strategy for fabricating high-performance perovskite photodetectors with MO-NP-modified ITO electrodes offers high compatibility with low-cost, flexible, and large-area electronics [62]. Hang et al. indicated that the Mg_2_Si/Si heterostructure, as a non-toxic, environmentally friendly semiconductor system, has significant potential for visible-to-near-infrared photodetection. Mg_2_Si/Si thin films were prepared by magnetron sputtering followed by annealing, and nanostructures were deposited on the Si substrate surface to enhance both film quality and photodetector performance. Mg_2_Si/Si heterojunction photodetectors, the structure of which is illustrated in Figure 8a, were fabricated based on n-type Si wafers and Mg_2_Si thin films grown on the Si substrates. Figure 8b presents the dark current characteristics of Mg_2_Si/Si heterojunction photodetectors prepared at different annealing temperatures for a fixed annealing duration of 8 h. Figure 8c shows the I-V curve measured in the dark as well as under illumination at wavelengths ranging from 405 nm to 1550 nm. Figure 8d,e display the responsivity (R) and specific detectivity (D*) of the Mg_2_Si/Si heterojunction photodetectors prepared at different annealing temperatures under a 1 V bias. As shown in Figure 8f, the device exhibits a response time of 1.61 ms at 1064 nm. The incorporation of nanostructures substantially improves the responsivity and specific detectivity of the photodetectors, offering a promising strategy for developing high-performance Mg_2_Si/Si-based optoelectronic devices.

Improved CH_3_NH_3_PbI_3_/Si heterojunction PDs were fabricated by passivating interfacial defects using a thin amorphous HfO_2_ layer grown via low-temperature atomic layer deposition. The results indicate that the HfO_2_ layer effectively passivates the surface defects of Si and moderately enhances the quality of the CH_3_NH_3_PbI_3_ thin film by increasing its grain size [64]. Cao et al. deposited ZnGa_2_O_4_ thin films on p-GaN epitaxial layers using pulsed laser deposition, achieving films with improved crystalline quality [65]. To form the heterojunction PD, 50 nm-thick Au electrodes were thermally evaporated onto both the p-GaN and ZnGa_2_O_4_ layers. The n-ZnGa_2_O_4_/p-GaN PD exhibited a response time of 39 ms and a recovery time of 30 ms. Based on these results, the band diagram of the n-ZnGa_2_O_4_/p-GaN heterojunction under negative bias was derived. Photogenerated carriers were generated in both the ZnGa_2_O_4_ layer and the GaN epitaxial layer. The photogenerated carriers were separated and pumped away by the built-in electric field and the large external electric field. Because the potential barrier of the valence band was only 0.36eV, the holes in the ZnGa_2_O_4_ layer transferred to the GaN layer easily and then flowed to the negative electrode with the holes in GaN. Meanwhile, the conduction band electrons in ZnGa_2_O_4_ flowed toward the positive electrode. Moreover, increasing the illumination intensity can fill the trap states, raising the quasi-Fermi level of electrons closer to the CB, and the device may then lose the gain initially governed by the presence of partially filled trap states [66]. This work contributes to the development of next-generation optoelectronic devices.

A nanostructured tin(IV) oxide (SnO_2_)/Si heterojunction UV photodetector was fabricated via pulsed laser deposition (PLD) under controlled laser pulse conditions. The photodetection mechanisms of the designed devices were systematically investigated with respect to multiple experimental parameters, including laser pulse conditions, spectral response, and incident optical power [67]. Zeng et al. constructed a Bi_2_Se_3_/ZnO nanowire array (NWA) heterojunction with type-I band alignment in a 2D material system, achieving a broadband photoresponse from the UV to the near-infrared region. In this work, a type-I band-aligned heterojunction was formed between 2D Bi_2_Se_3_ and ZnO NWAs. The resulting device not only exhibits a photoresponsivity of 0.15 A/W at 377 nm—which is over three times higher than that of pure ZnO nanowires (0.046 A/W)—but it also exhibits a broadband photoresponse covering the ultraviolet to near-infrared region. These findings suggest that the Bi_2_Se_3_/ZnO NWA type-I heterojunction can serve as a promising candidate for broadband photodetection applications [68]. Posakb et al. reported a novel MoS_2_/Co_3_O_4_ heterojunction based on bioplastic semiconductors, revealing an energy band mechanism influenced by carrier concentration. The study demonstrated the optimized device’s promising performance as a photodetector, thereby advancing the application of bioplastics in electronics [69].

P-N junction photodetectors are advancing beyond simple performance metrics toward functional integration. While type-II heterojunctions achieve exceptional responsivity via defect engineering, this often compromises speed and exacerbates persistent photoconductivity, a critical trade-off for real-world deployment. In contrast, self-powered designs prioritize energy efficiency but lag in sensitivity. A more transformative trend is emerging: sensing-computing fusion. Recent GaN-based and Ga_2_O_3_/GaN heterostructures demonstrate in-sensor processing-integrating detection, memory, and neuromorphic computation within a single diode. These smart detectors sacrifice marginal gain to enable on-chip intelligence, positioning them as key enablers for edge-AI vision systems. The central challenge now lies in merging high-fidelity detection with such functional versatility.

### 2.6. Perovskite Photodetectors

A PPD represents a modern optoelectronic device fabricated from perovskite-based compounds, most commonly organic-inorganic hybrid halide perovskites. By leveraging the photoelectric effect, it converts incoming light—covering ultraviolet, visible, and near-infrared wavelengths—into detectable electrical outputs such as current or voltage. With its exceptional photoresponsive properties, the PPD has established itself as a highly promising component for future optical sensing and imaging technologies [70]. Hybrid organic-inorganic perovskites have drawn significant interest in the field of optoelectronic devices due to their superior characteristics. Specifically, their favorable optical and electrical properties, including high optical absorption coefficients, high carrier mobility, and extended carrier diffusion lengths, offer substantial potential for advancing high-performance photodetectors. As an intrinsic physical characteristic of light, polarization carries and encodes rich optical information. Perovskite materials, featuring excellent photovoltaic performance, anisotropic crystal structures, and controllable oriented growth, are widely used in polarization-sensitive photodetectors. Despite the great potential of organic–inorganic perovskites for low-cost light-harvesting devices, the relatively low carrier mobilities of bulk perovskites remain a bottleneck for the performance of large-area devices, especially compared with the most advanced technologies [71]. Perovskite photodetectors have evolved into versatile optoelectronic devices with broad application prospects across the visible-to-near-infrared spectral range. They are widely used in imaging systems (e.g., medical imaging, night vision surveillance, and smartphone cameras), optical communication networks for high-speed signal detection, and environmental monitoring (including gas sensing and ultraviolet radiation detection), as well as emerging fields such as artificial-intelligence-enabled machine vision, flexible electronics, and wearable health monitors. Their exceptional photoelectric performance, low-cost fabrication, and compatibility with flexible substrates further expand their potential in next-generation optoelectronic technologies.

**Figure 9:**
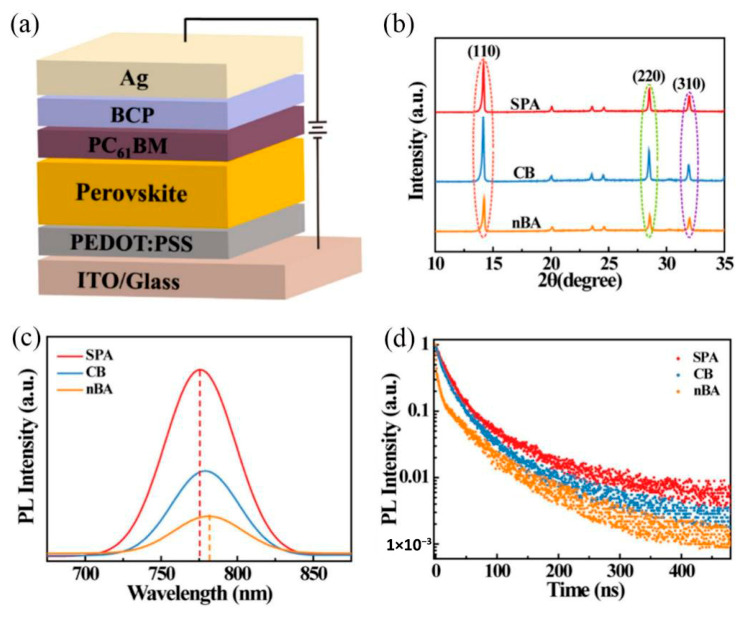
(**a**) Device structure of the PPDs. (**b**) XRD patterns of the perovskite films. (**c**) Steady-state photoluminescence (PL) spectra. (**d**) TRPL spectra [10]. Copyright 2021, ACS Publications.

Interface engineering was applied to a hybrid trilayer (TiO_2_/graphene oxide/perovskite) photodetector to improve crystallinity and achieve effective defect passivation. This study highlights the role of interface engineering in enhancing device performance through improved interfacial defect passivation and more efficient carrier transport [72]. Hong et al. noted that lead halide perovskite photodetectors hold great potential for broadband photodetection but face critical challenges, including ion migration and parasitic charge injection. They use an ultra-thin layer of FcPhc_2_ as a hole-blocking layer. FcPhc_2_ effectively inhibits I-oxidation induced by injected holes and reduces formed I_2_ on the perovskite surface, enhancing reverse bias stability [9]. Kong et al. noted that with advances in photodetector technology, integrating perovskite photodetectors into imaging sensors has become a key objective in optoelectronics. However, this effort is constrained by issues such as high energy consumption and pixel-to-pixel optoelectrical crosstalk. They developed a self-driven 10 × 10 perovskite photodiode blocking diode (PIN BD) photodetector array with high responsivity and detectivity and elucidated the mechanism by which PIN BD integration suppresses crosstalk [73]. Li et al. emphasized that fabricating high-quality perovskite films is a critical prerequisite for achieving superior performance in perovskite PPDs. In this work, a low-toxicity antisolvent SPA is employed to regulate film formation, resulting in a high photoresponsivity of 1.06 A/W at 532 nm. The polarity of the antisolvent is shown to influence both the quality of the perovskite film and device performance. Figure 9a presents the device structure of the fabricated PPDs, where ITO functions as the anode, and a bilayer consisting of PEDOT:PSS and phenyl-C_61_-butyric acid methyl ester/bathocuproine (PC_61_BM/BCP) serves as the HTL and ETL, respectively. The XRD patterns of perovskite films grown on PEDOT:PSS-coated ITO/glass substrates are illustrated in Figure 9b. Figure 9c shows that the SPA-processed perovskite (SPA-PSK) film exhibits the strongest photoluminescence (PL) emission, with a peak at 775.25 nm, and the CB-PSK and nBA-PSK films have drastically reduced PL intensities in comparison to the SPA-PSK film. The time-resolved photoluminescence (TRPL) decay curves in Figure 9d further confirm this result.

This study provides a valuable reference for preparing high-performance perovskite photodetectors.

**Figure 10:**
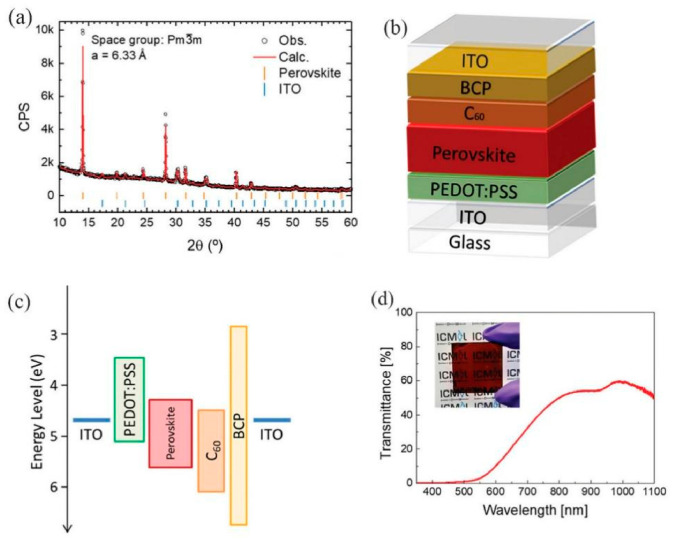
(**a**) X-ray diffraction pattern of MA_0.3_FA_0.7_Pb_0.5_Sn_0.5_I_3_ perovskite thin films grown on glass/ITO substrates modified with PEDOT:PSS. Experimental intensity data are indicated by open circles, while the red curve represents the Le Bail fit; vertical markers in distinct colors identify Bragg reflections of the perovskite phase and the underlying ITO phase. (**b**) Device structure schematic of the semitransparent perovskite photodetector. (**c**) Energy band diagram corresponding to the photodetector. (**d**) Transmittance spectra of the full device stack featuring a 200 nm-thick active layer. Inset: Optical photograph of the device placed over the logo of the research institute conducting this work [75]. Copyright 2022, Royal Society of Chemistry.

Liu et al. reported the electrical and structural characteristics of 50 nm-thick ZnO MSM UV photodetectors after proton irradiation at different temperatures. Irradiation induced the degradation of crystal quality, shifts in diffraction peaks, and changes in defect states. The observed persistent photoconductivity was attributed to surface traps and interactions with O_2_ molecules, with these defects exerting a long-term adverse effect on device performance. The proposed novel PM-PD structure is also compatible with potential 3D monolithic integration [74]. Arghanoon et al. fabricated semitransparent NIR perovskite photodetectors based on tin-lead (Sn-Pb) hybrid perovskites using very thin perovskite layers (200 nm) and transparent ITO electrodes. The performance of these devices positions them as promising candidates for NIR photodetection or bifacial device applications. XRD characterization of a MA_0.3_FA_0.7_Pb_0.5_Sn_0.5_I_3_ perovskite thin film is presented in Figure 10a. Photodetectors were fabricated using MA_0.3_FA_0.7_Pb_0.5_Sn_0.5_I_3_ perovskite thin films as the active layer, as shown in Figure 10b. The corresponding energy band diagram is illustrated in Figure 10c. Figure 10d shows the measured transmittance of the full device stack with the 200 nm perovskite film.

Perovskite photodetectors excel in solution-processed versatility but are fundamentally constrained by ionic instability and high dark current. Recent advances pivot decisively to interface engineering—from self-assembled monolayers to graphene heterostructures—to suppress defects and reconcile the mobility–lifetime trade-off. A paradigm shift emerges: leveraging ion migration for narrowband detection and prioritizing interfacial synergy over bulk crystal perfection. Lead-free variants mitigate toxicity but lag in stability. The field now focuses on stabilizing low-light, self-powered operation for practical deployment.

### 2.7. Hybrid Silicon–Polymer Photodetectors

A hybrid silicon-polymer photodetector (HSPPD) is an advanced optoelectronic device that integrates crystalline silicon–a mature semiconductor with excellent charge transport properties–with functional organic polymers, which offer tunable optical absorption and low-cost processability, into a single architecture. Unlike pure silicon or polymer photodetectors, the HSPPD combines the complementary advantages of both material systems to achieve high-performance light detection across UV, visible, and NIR spectral ranges. Hybrid design typically features a silicon substrate modified with polymer layers-such as conjugated polymers or donor-acceptor blends-or incorporates a heterojunction structure in which silicon and polymer components form an interface for efficient light-to-electricity conversion [76]. An HSPPD integrates crystalline silicon-which has strong charge transport properties, with functional organic polymers that offer tunable light absorption and low-cost fabrication. Unlike conventional silicon- or polymer-based PPDs, HSPPDs enable highly effective photoresponse across the ultraviolet to near-infrared wavelength range. Standard designs often incorporate silicon substrates with polymer layers or silicon–polymer heterojunctions to convert light into electrical signals. Outside the realm of photodetection, silicon and its oxides are widely employed in biomedical fields such as tissue regeneration and the controlled release of therapeutics, benefiting from their excellent biocompatibility, customizable surface properties, and capacity to be metabolized into harmless silicic acid in biological environments [77]. In fiber-optic communication systems, HSPPDs can receive signals quickly, achieving data rates of up to 10 Gbps thanks to their fast response time. For environmental sensing applications, these detectors can identify ultraviolet light, toxic gases, and airborne contaminants by using polymer coatings that are designed to absorb specific wavelengths. In addition, b-Si material possesses surface properties that can minimize reflection to the greatest extent, making it an ideal candidate for constructing highly responsive photodetectors. This innovative device opens up new sensing applications in areas such as night vision, movement detection, and biological sensing [78].

Hybrid Si-polymer photodetectors merge CMOS compatibility with organic flexibility, yet performance is interface-limited. Critical advances center on conformal interfacial engineering—e.g., oCVD PEDOT on black-Si-to reconcile Si’s high surface recombination with the polymers’ low mobility. Type-II heterojunctions enable responsivities exceeding 4000 A/W and bias-switchable multimodal detection unattainable in conventional Si diodes. However, polymer instability under bias and environmental conditions remains the decisive bottleneck, shifting the focus from initial performance to interface durability.

### 2.8. Broadband Photodetectors

A broadband photodetector can detect light across a broad wavelength range. As a high-performance optoelectronic device, its detection spectrum generally extends from the UV and visible regions into the NIR regime, approximately from 200 nm to 2500 nm, and can sometimes extend into the mid-infrared region. In contrast to narrowband detectors, which operate only within a limited wavelength band, broadband detectors achieve consistent sensitivity across a broad range. This is accomplished by incorporating specially chosen materials, such as perovskites, silicon–polymer blends, or 2D materials-into carefully engineered device structures. The main task of these detectors is to convert incoming light into electrical signals, such as changes in current or voltage, with high efficiency across all targeted wavelengths. Perovskite-based detectors function by effectively converting incoming light into excitons, which then dissociate into mobile charges collected at the contacts. To achieve broadband photodetection, one approach is to employ absorber materials with intrinsically broad spectral response. Another strategy utilizes a stacked architecture that incorporates complementary light-absorbing layers, for instance, combining a UV-selective organic semiconductor with a perovskite active layer sensitive to near-infrared wavelengths-thereby enabling detection over an extended spectral range [79]. Upon illumination, photons of varying wavelengths are captured by their respective active layers within the device, leading to the formation of electron–hole pairs (excitons). Modern design strategies, including the use of nanostructured surfaces or plasmonic effects, can further enhance light absorption and confinement, thereby achieving high photoresponsivity over a wide spectral band [80]. Broadband photodetectors find versatile applications across multiple domains. In remote sensing, they enable monitoring crop health and tracking environmental pollutants. For medical imaging, these devices support biomedical fluorescence imaging and tissue spectroscopy, aiding in disease diagnosis. Additionally, they serve as key components in wavelength-division multiplexing (WDM) in optical communication systems, enabling high-speed, multi-channel data transmission. In industrial settings, they are applied to defect detection in semiconductors and food packaging. Security surveillance benefits from the use of broadband detectors in UV and NIR cameras for continuous monitoring. Additionally, broadband photodetectors are employed in scientific research, including spectroscopy and quantum optics experiments, as well as in wearable health devices for physiological signal sensing.

**Figure 11:**
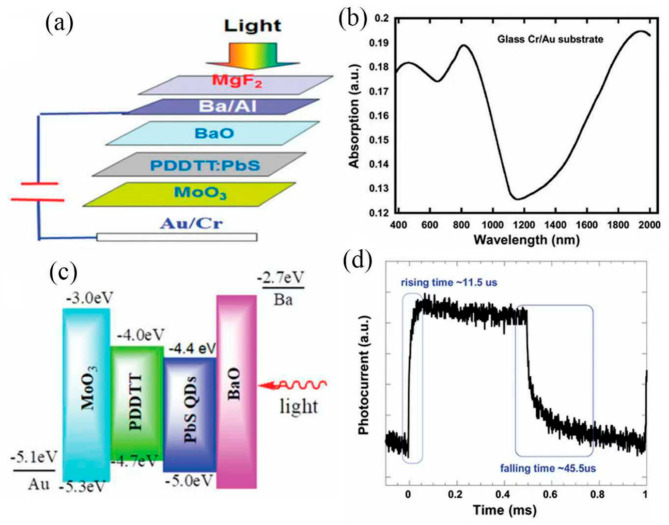
(**a**) Device architecture of the broadband photodetector; (**b**) absorption spectrum of the glass/Cr/Au thin film; (**c**) LUMO and HOMO energy levels of MoO_3_, PDDTT, PbS QDs, and BaO, together with the work functions of the Au and Ba electrodes; (**d**) photocurrent response time of the broadband photodetector [84]. Copyright 2021, Royal Society of Chemistry.

Yang et al. proposed a self-powered UV-Vis-NIR photodetector constructed from β-Bi_2_O_3_ nanoparticles. The inherent defects produced during low-temperature thermal oxidation of bismuth endow the material with efficient light absorption across the entire UV-Vis-NIR spectral range, demonstrating the potential of β-Bi_2_O_3_ for high-efficiency broadband photodetectors and offering a novel strategy for constructing such devices [81]. Xiao et al. conducted a numerical investigation on photodetectors with a transparent conductive oxide (TCO)/semiconductor/metal structure, focusing particularly on devices with a roughened interface; they analyzed the influence of interface roughness on hot-electron injection efficiency [82]. Xin et al. noted that rGO, a two-dimensional material with a tunable bandgap, holds significant potential for broadband photodetection systems. To enhance the interfacial contact area of heterojunctions in silicon nanowire (Si NW)-based photodetectors, a sequential process was employed to fabricate a [Si NWs/rGO]/rGO heterojunction as the active layer. This approach yielded photodetectors with high specific detectivity and responsivity, and the underlying physical mechanisms were also discussed [83]. Sheng et al. reported solution-processed broadband photodetectors (PDs) featuring an “inverted” vertical photodiode structure without transparent conductive oxide electrodes. The devices were fabricated from bulk heterojunction composites comprising a low-optical-gap conjugated polymer and highly conductive PbS quantum dots, demonstrating strong performance across the UV-visible-to-infrared range. Figure 11a illustrates the device architecture of the broadband PDs. A smooth Au layer, which typically reflects around 30% of incident light, was employed as the reflective electrode. Figure 11b shows the absorption spectrum of the glass/Cr/Au thin film. Figure 11c illustrates the lowest unoccupied molecular orbital (LUMO) and highest occupied molecular orbital (HOMO) energy levels of MoO_3_, PDDTT, PbS QDs, and BaO, alongside the work functions of the Au and Ba electrodes. The transient photocurrents of the broadband PDs are displayed in Figure 11d, where the rise time is defined as the duration taken for the output signal to increase from 10% to 90% of its saturated photocurrent value.

**Figure 12:**
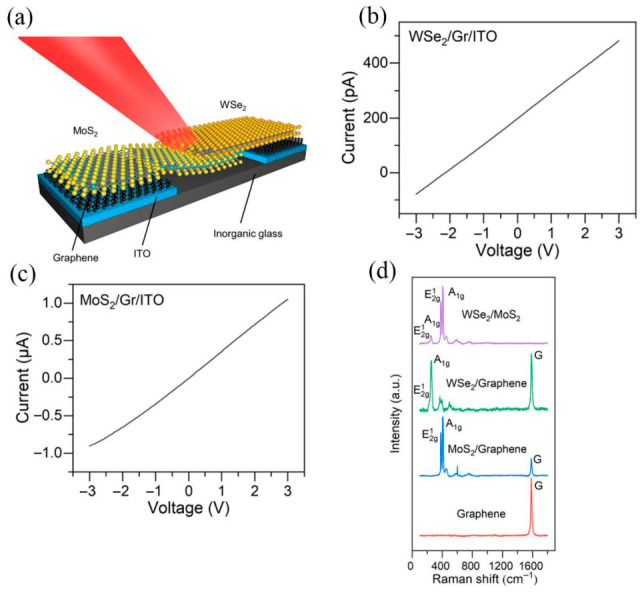
Device architecture and characterization. (**a**) Schematic and optical image of the WSe_2_/MoS_2_ heterostructure with Gr/ITO hybrid electrodes. (**b**) Gr/ITO hybrid electrode configuration. (**c**) EQE curves of the WSe_2_/MoS_2_ heterostructure with Gr/ITO hybrid electrodes and with bare ITO electrodes. (**d**) Raman spectra of multilayer graphene, WSe_2_, MoS_2_, and their overlapped regions [86]. Copyright 2022, AIP Publishing.

Basanta et al. noted that broadband photodetectors based on two-dimensional semiconductors have attracted significant research interest in the field of optoelectronics. The report presents a high-performance photodetector based on rGO-decorated p-γ-In_2_Se_3_/n-Si heterostructures, which exhibits enhanced responsivity, high sensitivity, fast response/recovery times, and a broadband response [85]. Xiao et al. pointed out that nanoscale photodetectors are essential for the development of intelligent imaging systems and wireless communication technologies. Although the construction of vdWs heterostructures based on two-dimensional transition metal dichalcogenides shows great potential, the performance of such devices is frequently restricted by contact problems. In this work, a WSe_2_/MoS_2_ heterostructure photodetector incorporating graphene/ITO hybrid electrodes was prepared. This design reduces lattice damage and facilitates efficient carrier transport, resulting in a device with high responsivity and detectivity, as well as a fast response to light from ultraviolet to near-infrared. Figure 12a illustrates a schematic and an optical image of the WSe_2_/MoS_2_ heterostructure integrated with Gr/ITO hybrid electrodes on a glass substrate. The introduction of the hybrid electrodes significantly enhanced the overall photoresponse performance, as shown in Figure 12b. Figure 12c compares the EQE of the heterostructure equipped with Gr/ITO hybrid electrodes versus bare ITO electrodes. While both configurations exhibit similar wavelength-dependent trends, the device with hybrid electrodes shows a remarkable improvement in EQE across the 405–980 nm laser wavelength range. Figure 12d presents the Raman spectra of the WSe_2_/MoS_2_ heterostructure with Gr/ITO hybrid electrodes, obtained using a HORIBA iHR550 imaging spectrometer with a 532 nm laser source.

Broadband photodetectors (BPDs) spanning the visible to mid-infrared spectral range outperform conventional narrowband silicon/InGaAs devices in spectral coverage and cost. Recent advances have addressed long-standing flaws: solution-processed Cu_2_CdSnSe_4_ eliminates the need for costly vacuum fabrication; PbS quantum dots match commercial InGaAs performance and extend detection to 2330 nm. Emerging 2D/halide perovskite heterojunctions and angular-selective microstructures further enable ultra-sensitive mid-infrared sensing and low-noise operation. Poor batch uniformity in solution-processed devices, quantum dot lead toxicity, and high-precision fabrication requirements for mid-infrared structures still hinder large-scale deployment.

### 2.9. Solar-Blind Photodetectors

A solar-blind photodetector is a specialized optoelectronic component that detects UV radiation in the solar-blind region (200–280 nm). In this spectral band, solar irradiation is fully absorbed by the Earth’s ozone layer. Unlike conventional UV detectors, it operates exclusively within this ozone-shielded band, thereby eliminating interference from solar background radiation and visible light. Typically fabricated using wide-bandgap semiconductors such as AlGaN, MgZnO, or diamond [87], the device converts incident solar-blind UV photons into measurable electrical signals-such as photocurrent or voltage-with high selectivity, making it ideal for low-noise detection in outdoor and daytime environments. Solar-blind ultraviolet photodetectors offer several advantages, including low false-alarm rates, high sensitivity to weak signals, and excellent signal-to-noise ratios. Gallium-based alloys such as AlGaN and Ga_2_O_3_ are widely utilized as functional materials, particularly in the role of channel semiconductors, and have been the subject of extensive research for decades. Solar-blind photodetectors play a critical role in a variety of practical applications. Their defining characteristic is the ability to detect solar-blind UV radiation, which is naturally absent in sunlight, thereby eliminating solar interference. This unique capability makes them highly valuable across multiple fields. They are employed for defect inspection in semiconductor wafers and other UV-sensitive materials. For civilian purposes, solar-blind photodetectors are integrated into fire alarm systems installed in buildings and public spaces, enabling rapid detection of UV signatures from flames. Furthermore, they support anti-counterfeiting efforts by detecting UV-responsive security features on banknotes and official documents [88]. All these applications are based on their properties.

**Figure 13:**
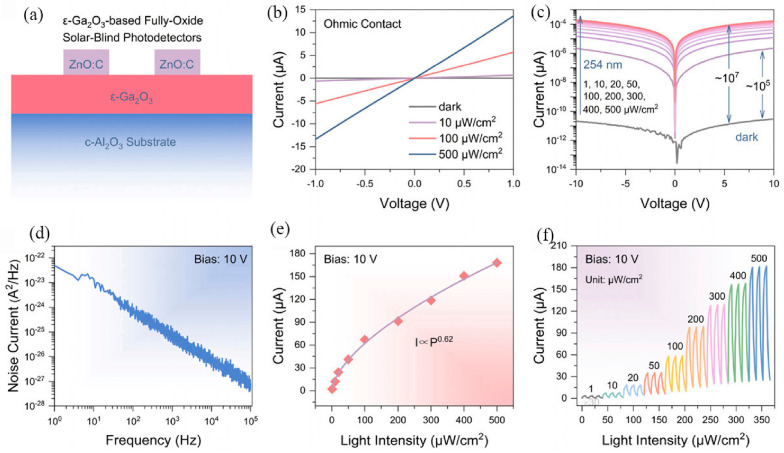
(**a**) Schematic structure of the ε-Ga_2_O_3_-based all-oxide solar-blind deep-ultraviolet photodetector. (**b**) Criteria confirming Ohmic contact between the ZnO:C transparent conductive film and the ε-Ga_2_O_3_ layer. (**c**) Current-voltage (I-V) characteristics (log-scale) under dark conditions and 254 nm illumination. (**d**) Noise spectral density of the device. (**e**) Photocurrent as a function of incident light intensity. (**f**) Transient photoresponse of the device under 254 nm illumination at different light intensities [89]. Copyright 2024, AIP Publishing.

Li et al. studied carbon doping as a means to improve the electrical properties of ZnO films for transparent conductivity. They found that ZnO:C films preserve high structural quality and surface morphology, even as defect density increases with higher C doping. Carbon doping does not degrade visible-light transmittance and gradually widens the optical bandgap, consistent with the Burstein–Moss effect. Figure 13a illustrates the structure of a transparent SBPD based on ε-Ga_2_O_3_, in which ZnO:C transparent conductive films serve as the electrode material; the electrode channel dimensions are 20 × 500 μm^2^. Figure 13b depicts the contact behavior between the ZnO:C electrode and the ε-Ga_2_O_3_ film, confirming a typical Ohmic contact and verifying that the fabricated device operates as a photoconductor. Despite the Ohmic nature of the contact, the device exhibits low dark current and low noise current, as shown in Figure 13c and Figure 13d, respectively. Under increasing illumination intensity at 254 nm, the photocurrent rises significantly, reaching values on the order of 10^−6^–10^−4^ A, while the photocurrent-to-dark-current ratio approaches 10^5^–10^7^. This demonstrates the high sensitivity of the transparent photodetector to solar-blind deep-ultraviolet light. The photocurrent increases with light intensity (Figure 13e), following a power-law relationship with an exponent of 0.62. Figure 13f presents the time-dependent photoresponse (I-t) curves of the device under a 10 V bias and varying light intensities.

**Figure 14:**
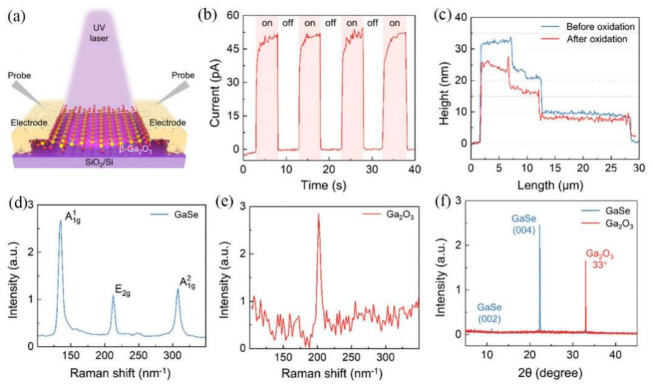
(**a**) Schematic of the MSM architecture used for testing β-Ga_2_O_3_-based solar-blind UV photodetectors. (**b**) Current-time (I-t) curve of the device based on 12 nm β-Ga_2_O_3_. (**c**) Thickness of the nanosheets before and after oxidation, measured via atomic force microscopy (AFM) along the lines indicated in (**b**). (**d**) Raman spectrum of the pristine GaSe nanosheets. (**e**) Raman spectrum of the oxidized β-Ga_2_O_3_ nanosheets. (**f**) XRD spectra of the nanosheets before oxidation (red line) and after oxidation (blue line) [90]. Copyright 2024, Royal Society of Chemistry.

Hu et al. emphasized that effective thermal management is essential for the stable, long-term operation of both electronic power devices and Ga_2_O_3_-based UV photodetectors, as Ga_2_O_3_‘s low thermal conductivity leads to heat accumulation and associated performance degradation. In this study, quasi-two-dimensional β-Ga_2_O_3_ layers of varying thicknesses were synthesized from two-dimensional GaSe nanoflakes. This synthesis approach limits the thickness of the β-Ga2O3 formed by oxidation, enhances the specific surface area, suppresses hot-carrier accumulation, and offers a potential solution to the thermal challenges of wide-bandgap optoelectronic devices. Monochromatic light tests were performed using a probe station. Tungsten-steel probes were placed in contact with the two electrodes of the material, and a test voltage was applied through the probes (Figure 14a). Figure 14b displays time-dependent photocurrent responses for the 12 nm detector tested using monochromatic light. The observed reduction in thickness suggests a structural transformation in which interlayer van der Waals interactions with relatively large spacing are converted into strong covalent bonds with a more compact stacking configuration, indicative of the structural change induced by oxidation (Figure 14c). Raman spectroscopy was further employed to investigate the structural transformation of GaSe samples during oxidation (Figure 14d). The pristine GaSe sample showed distinct Raman peaks at A^1^_1g_ (134.7 cm^−1^), E_2g_ (212 cm^−1^), and A^1^_2g_ (307.7 cm^−1^), which confirms its high crystalline quality. In contrast, the oxidized GaSe sample exhibited a Raman peak at 201.8 cm^−1^. (Figure 14e), which is characteristic of β-Ga_2_O_3_, confirming its formation during the oxidation process. XRD results (Figure 14f) further indicated significant structural changes after oxidation.

Solar-blind photodetectors (SBPDs) are critical for fire warning and environmental monitoring, as they outperform conventional UV detectors in terms of immunity to solar background radiation. While traditional Ga_2_O_3_-based devices suffer from high dark current and low responsivity, 2025–2026 advances have unlocked multifunctional, self-powered solutions: dual-heterojunction engineering boosts responsivity to 183 mA/W; WS_2_/GaPS_4_ heterostructures enable polarization imaging; bio-hydrovoltaic integration realizes maintenance-free operation in harsh environments. Fully transparent, wide-temperature (100–450 K) devices further expand the application scenarios. Ga_2_O_3_ thin-film uniformity remains poor; 2D heterostructures have low yield; and bio-hydrovoltaic modules lack long-term stability, hindering large-scale deployment.

### 2.10. Graphene Photodetector

A graphene photodetector is an advanced optoelectronic device that employs graphene-a 2D monolayer carbon material-as its active light-detection layer. Graphene’s unique electronic properties, including its zero bandgap, high carrier mobility, and broadband spectral absorption, enable the device to detect light across a wide range of wavelengths, from UV and visible to NIR and even mid-infrared (MIR) regions (200 nm–5 μm) [91]. Unlike conventional semiconductor photodetectors, such as silicon or III-V compounds, graphene photodetectors typically operate via photo-thermoelectric or photoconductive mechanisms, converting incident photons into measurable electrical signals with high speed and versatility. Graphene’s flexibility and high optical transparency enable its integration into wearable electronics, such as flexible physiological monitors, as well as smart technologies, including see-through photodetectors for smart windows and solar energy systems. These characteristics also drive progress in scientific domains such as ultrafast optical studies and quantum communication.

**Figure 15:**
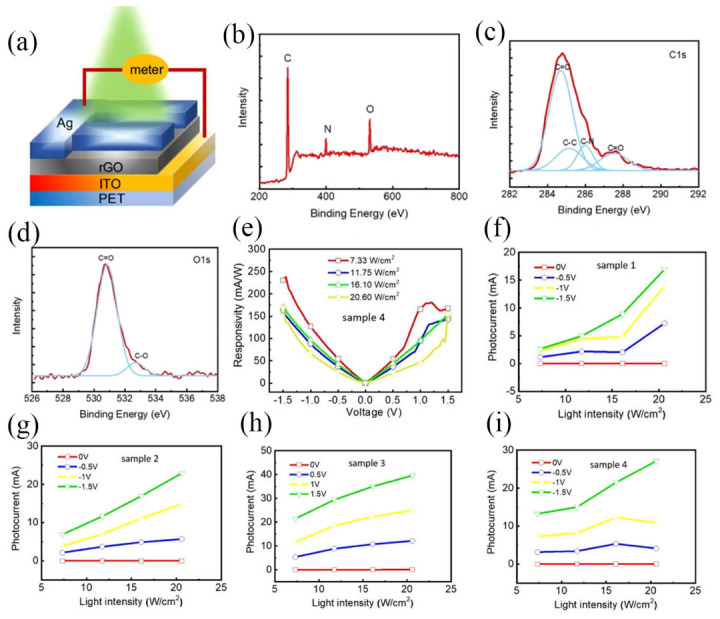
(**a**) Schematic cross-sectional view of a flexible, semi-transparent rGO photodetector. (**b**) XPS spectrum of block-copolymer-modified rGO flakes. High-resolution XPS spectra of (**c**) the C 1s region and (**d**) the O 1s region. (**e**) Responsivity as a function of bias voltage for samples 1–4. (**f**–**i**) Photocurrent as a function of light power density under different bias voltages for sample 1, sample 2, sample 3, and sample 4, respectively. The photocurrent is defined as $I_{p}=I_{L}-I_{D}$, where $I_{p}$ is the photocurrent, $I_{L}$ is the current under illumination, and $I_{D}$ is the dark current. Notably, even under a low bias of −1.0 V and a light intensity of 20.60 W/cm^2^, samples 1, 2, 3, and 4 exhibit excellent photocurrent values of 13.93 mA, 14.85 mA, 25.02 mA, and 10.88 mA, respectively [95]. Copyright 2021, Optical Publishing Group.

The ability of graphene to convert optical signals into electrical signals plays a crucial role in advancing modern information technology. This study clarifies the mechanism of photoelectric conversion in high-quality graphene, presents a gate-tunable graphene photodetector with wide bandwidth, and demonstrates the instantaneous extraction of photocurrent along with its electrical tunability-thereby effectively linking ultrafast optical science with practical device engineering [92]. Elsayed et al. developed a GO/poly-3-methylaniline (P3MA) photodetector for broadband light detection. The study details the synthesis procedure, characterization of material properties, and evaluation of the photodetector’s performance under various illumination conditions, highlighting its potential for future applications in light detection [93]. In a separate study, graphene oxide films (GOFs) were used as the active material in an infrared photodetector configuration, and the temperature dependence of the films’ electrical conductivity was systematically investigated. This research provides a foundation for the further development of highly conductive graphene oxide films through scalable preparation methods [94]. As noted by Tu et al., the growing demand for high-performance optoelectronic systems has driven the development of 2D graphene-based photodetectors. Yet, traditional manufacturing techniques for these devices are often expensive and complex to operate. To resolve this, a semi-transparent rGO photodetector with ultra-low power consumption was prepared on a PET substrate via a simple process, demonstrating promising performance and great potential for integration into future wearable devices. Figure 15a presents a schematic diagram of the vertical Ag/rGO/ITO/PET photodetector structure. XPS, as shown in Figure 15b, was used to analyze the elemental composition and bonding environment of the rGO flakes. The peaks detected at 285 eV, 400 eV, and 532 eV correspond to C 1s, N 1s, and O 1s, respectively, confirming that the block-copolymer-modified rGO flakes were successfully coated on the substrate. The deconvoluted C 1s XPS spectrum (Figure 15c) clearly shows the carbon bonding configurations in various functional groups of the rGO film, including C=C (284.6 eV), C-C (285.1 eV), C-N (285.9 eV), and C=O (287.6 eV). Likewise, the O 1s spectrum (Figure 15d) can be decomposed into two components: C=O at 531.0 eV and C-O at 532.6 eV. The responsivity of samples 1 to 4 as a function of bias voltage is assessed in Figure 15e. Figure 15f–i display the photocurrent of sample 1, sample 2, sample 3, and sample 4 as a function of light power density under different bias voltages, respectively.

Graphene photodetectors excel in ultrafast speed and broadband response, outperforming conventional semiconductors in on-chip integration potential, but suffer from intrinsic low light absorption and low responsivity. Advances have addressed from 2025 to 2026 have addressed these flaws: CsPbBr_3_ quantum dot sensitization stabilizes photocurrent and boosts response speed; nonvolatile p-i-n homojunctions enable zero-bias operation with 17 GHz bandwidth; and low-temperature graphene/copper structures achieve a record responsivity of 666.95 mA/W. Emerging van der Waals heterojunctions further enable neuromorphic photodetection. Large-area, uniform graphene growth remains challenging; hybrid heterostructures exhibit poor interface stability, and high fabrication costs still hinder mass production.

### 2.11. Quantum Dot Photodetectors

Quantum dot photodetectors (QDPDs) constitute a category of optoelectronic devices in which semiconductor nanocrystals, commonly termed quantum dots, serve as the light-sensitive core. These nanocrystals display tunable electronic properties determined by their size and operate by converting incident photons into measurable electrical signals. Their operational mechanism is primarily governed by the quantum confinement effect [96]. The optical response range of these devices can be precisely tuned from the ultraviolet to the infrared spectrum by varying the size of the quantum dots. Additionally, the exceptionally high absorption cross-section of quantum dots contributes to superior responsivity and enhanced detection performance. From a fabrication perspective, solution-processable techniques such as spin coating and inkjet printing enable low-cost, large-area production on flexible substrates. Quantum dot photodetectors also maintain stable operation under ambient conditions, eliminating the need for complex cooling systems and facilitating their integration into practical applications. These encompass high-sensitivity, fluorescence-based biomedical imaging, environmental sensing of trace gases and pollutants, high-speed optical communication receivers, low-light machine vision for autonomous systems, and infrared surveillance and missile alert systems in the defense and aerospace sectors [97].

**Figure 16:**
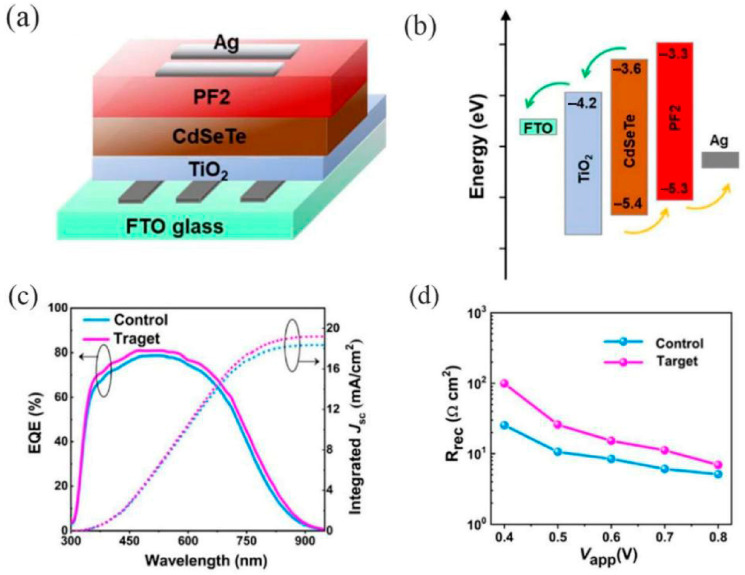
(**a**) Schematic structure of the self-powered broadband photodetector. (**b**) Energy band diagram and selected polymer hole-transport material (PF2) of the photodetector, along with the molecular structure of PF2. (**c**) EQE spectra and integrated short-circuit current density (J_sc_) of champion solar cells based on control and target CdSeTe QDs under standard AM 1.5 G illumination (100 mW cm^−2^). (**d**) R_rec_ as a function of applied bias voltage [99]. Copyright 2025, Royal Society of Chemistry.

A method for modulating repulsive interactions between surface ligands was developed. It was based on forming tight ion pairs with cationic surfactants to shift the colloidal stabilization of metal chalcogenide complex (MCC)-capped PbS quantum dots from long-range electrostatic to short-range steric mechanisms. This work demonstrates that ligand engineering can substantially enhance charge transfer, thereby improving the responsivity and detectivity of photoelectronic devices [98]. Sun et al. emphasized that efficient carrier transport within chalcogenide QDs is essential for high-performance optoelectronic operation. To examine the effect of in situ fluorination on carrier extraction and transfer in CdSeTe QDs, self-powered photodetectors with the structure ITO/TiO_2_/CdSeTe QDs/PF_2_/Ag were fabricated (Figure 16a). Figure 16b presents the corresponding band energy diagram and proposed charge transfer pathways. Under illumination conditions, the excitons generated from the CdSeTe QD absorbers then diffuse to the interface between the donor and acceptor, where they can dissociate into free charge carriers, followed by the charge carriers diffusing to the appropriate electrodes through the charge transfer layers under the built-in electric field. Moreover, using CdSeTe QDs with varying sizes and ligand treatments allows for controlling their electrical properties, including the Schottky barrier height and, therefore, the photocurrent [14]. The EQE spectra and integrated short-circuit current density (J_sc_) shown in Figure 16c confirm enhanced photocurrent generation in the target QD devices. As illustrated in Figure 16d, the recombination resistance (R_rec_) of samples containing the target QDs is significantly higher than that of the control sample under various bias voltages. This study introduces an in situ fluorination treatment using benzoyl fluoride to eliminate bulk traps in metal chalcogenide QDs, thereby accelerating carrier transport. Experimental results obtained from two types of photoelectrical conversion devices highlight the benefits of this treatment, offering promising prospects for the practical application of QD technologies.

Quantum dot (QD) photodetectors outperform conventional InGaAs devices in spectral tunability, solution processability and cost, but are limited by high dark current and material toxicity. Advances from 2025 to 2026 have delivered transformative performance: monomer-assisted PbS QDs achieve a record-low dark current of 21 nA/cm^2^ and extend detection to 2330 nm; and non-toxic Ag_2_Te QDs enable 3–5 μm mid-infrared sensing for body temperature monitoring. III-V QD-silicon photonic monolithic integration and water-mediated liquid QDs further expand application scenarios. Lead toxicity of PbS QDs, poor batch uniformity of solution-processed films, and limited long-term stability under ambient conditions remain major barriers to large-scale commercialization.

### 2.12. Other Photodetectors

Photodetectors are fundamental optoelectronic devices that convert incident light-spanning ultraviolet to infrared wavelengths-into measurable electrical signals, such as photocurrent or voltage. These devices encompass diverse types, including silicon-based, perovskite, graphene, hybrid silicon-polymer, solar-blind, and hybrid graphene/PbS quantum dot detectors, each distinguished by their active materials (e.g., semiconductors, two-dimensional materials, quantum dots, or composites) and structural designs. Their core function lies in sensing light intensity, wavelength, or phase within specific spectral bands. The operation of photodetectors uniformly follows three key steps: light absorption by materials with appropriate bandgaps or absorption coefficients; the generation of electron–hole pairs (excitons); and the dissociation of excitons into free charge carriers via built-in electric fields-from heterojunctions or P-N junctions or an external bias. The photoinduced charge carriers then travel through high-mobility channels to reach the electrodes, generating electrical outputs that correspond to the properties of the incoming light. Different material characteristics-such as graphene’s gapless electronic structure [100] and the tunable bandgap of PbS quantum dots-influence spectral range and response speed while the fundamental photoconversion process remains unchanged. In summary, photodetectors offer a combination of attractive attributes: broad spectral sensitivity from the UV [101] to visible and infrared regions; excellent performance metrics including picosecond-level response speeds (e.g., in graphene-based devices), responsivities above 1 A/W (as seen in perovskite and hybrid types), and very low dark current; diverse fabrication options ranging from low-cost solution-processing (for perovskites and PbS QDs) to conventional silicon microfabrication; and versatile designs compatible with both rigid and flexible platforms, which are further supported by their low power consumption and compact size.

Trujillo et al. employed graphene oxide films (GOFs) as the active material in an infrared photodetector configuration and systematically investigated the temperature dependence of the films’ electrical conductivity. This study lays the groundwork for further development of highly conductive graphene oxide films in scalable production [94]. Krystian et al. synthesized bismuth sulfoiodide (BiSI) nanorods via a low-temperature (393 K) wet chemical method. The one-dimensional crystalline structure of the BiSI nanorods was confirmed by high-resolution transmission electron microscopy (HRTEM). Morphology and chemical composition were examined using scanning electron microscopy (SEM) and energy-dispersive X-ray spectroscopy (EDS), respectively [102]. Amira et al. described the fabrication of freestanding rolled graphene oxide (roll GO) and polypyrrole (Ppy) composites using a modified Hummers’ method and oxidative polymerization. Overall, the results indicate that the prepared freestanding Ppy/roll GO/tape photodetector exhibits high potential for operation in the 340–730 nm optical range and may be suitable for industrial applications [103]. Zheng et al. fabricated an infrared photodetector based on an IGZO/PbSe CQD heterojunction. The PbSe CQDs were surface-capped with one of three ligands: MPA, TBAI, or EDT. The photodetection performance of the resulting devices was systematically studied and compared to identify the optimal surface ligand for PbSe CQDs in photodetection. This work provides practical and fundamental insights to guide the optoelectronic applications of PbSe CQDs [56].

A straightforward solvothermal technique was used to prepare tin monosulfide (SnS) nanosheets bonded with RGO. The resulting two-dimensional SnS nanosheets are uniformly layered on folded RGO, and a PET-based flexible photodetector fabricated from this hybrid exhibits a good optical response under visible light. Moreover, the SnS-RGO hybrid nanosheets exhibit high photocatalytic activity for the degradation of methylene blue. The near-complete degradation of methylene blue within 1 h indicates that SnS-RGO nanosheets are promising candidates for high-performance photocatalysts [104]. Wasan et al. detailed the fabrication and performance analysis of a photodetector based on a porous silicon (PS) structure embedded with indium oxide nanoparticles (In_2_O_3_ NPs). The In_2_O_3_ NPs were synthesized via pulsed laser ablation in ethanol (PLAL), while the porous silicon substrate was produced by photo-assisted electrochemical etching. The optical, structural, and electrical properties of the In_2_O_3_ NPs/PS devices were studied, with particular emphasis on their variation with laser energy [105]. Abinash et al. demonstrated the optical, electrical, and photoresponsivity performance of V_1−x_Mo_x_Se_2_ (x = 0, 0.05, 0.10, 0.15, 0.20) nanocomposites, synthesized via a facile hydrothermal method with tunable vanadium and molybdenum contents. Time-dependent photoresponse measurements revealed a high I_on_/I_off_ ratio for the VSe_2_ sample, highlighting its potential for fabricating sensitive photodetectors [106]. To address this, Sharafadeen et al. developed an engineered Ti_3_C_2_T_x_ Mxene (denoted FBT) co-modified with iron and boron for the selective oxygen evolution reaction. Boron incorporation was found to influence the growth of ZIF-67 on the Mxene surface. Both FBT and ZIF-67/FBT exhibited low overpotentials and good stability in alkaline seawater, expanding the potential of Mxenes as efficient OER catalysts for seawater splitting and related applications [107].

This study presents a method for fabricating vertical single-nanowire devices through targeted electrical contact formation to isolated nanowires within high-density arrays. Although nanowire-based optoelectronic and electronic systems exhibit clear advantages over traditional bulk materials, their full potential for ultra-high-resolution applications requires precise electrical interfacing at the individual nanowire level. The developed approach is compared with existing planarization techniques, yielding devices with notably improved ideality factors, detectivity, and photocurrent density. These enhancements facilitate the deployment of such devices in fields that demand ultra-high-resolution capabilities, including photodetection and advanced display technologies [108]. Sun et al. noted that tungsten oxide (WO_3_), a representative n-type semiconductor, holds significant promise for applications in photoelectronics and energy technologies. In this study, WO_3_ thin films were prepared by atmospheric-pressure spatial chemical vapor deposition (AP-SCVD) without post-deposition annealing. The resulting films showed strong performance across three different photoresponsive functions, underscoring the advantages of the AP-SCVD method for photoresponsive applications [109]. Hala et al. highlighted that while solar cells are critical renewable energy sources, metal oxide nanoparticles, such as CuO and Cu_2_O, also exhibit notable photoresponsivity advantages for related optoelectronic applications. They synthesized a CuO:Cu_2_O composite nanoparticle photodetector deposited on porous silicon, forming a type-II heterojunction suitable for photovoltaic applications [110]. Swati et al. pointed out that metal oxides are ideal materials for fabricating high-performance photodetectors. While n-type oxides have been widely studied, p-type oxides such as NiO remain less explored. Forming heterojunctions between p-type NiO and n-type ITO enhances detection performance. The effects of oxygen-to-argon ratios on NiO films and detector performance were investigated, paving the way for wide-bandgap metal oxide heterojunction photodetectors [111].

Solar-blind UV polarization detection and imaging are widely applied. They are critical for next-generation deep-UV optoelectronic systems, and β-Ga_2_O_3_ stands out as an ideal material candidate for enabling these functions. This study reports a method for preparing two-dimensional β-Ga_2_O_3_ flakes via liquid metal-assisted exfoliation. The resulting photodetector exhibits an ultrafast response, a high polarization photoresponse anisotropy ratio, and enables one-step, high-resolution polarization imaging in the solar-blind UV band [112]. Darragh et al. emphasized that metal-oxide thin films occupy a critical position in modern technology, including transistors and optoelectronic devices. Among them, n-type semiconducting zinc oxide (ZnO) possesses particularly useful properties. This review focuses on the unique characteristics of ZnO and related compound semiconducting oxides, discussing their applications and their impact on sustainable optoelectronics [113]. Karagiorgis et al. presented flexible and transparent photodetectors fabricated from PEDOT:PSS:Ag nanowire-based nanofibers and ZnO nanowires on a transparent, degradable cellulose acetate substrate. The developed photodetectors demonstrate high responsivity and stability, and are environmentally friendly due to their ability to degrade naturally at end-of-life [114]. Tsukruk et al. pointed out that n-type ZnO nanoparticles are widely used in sensing applications. In their work, they further demonstrated the high-resolution fabrication of noble metal-doped ZnO conductive channels via engraving transfer printing combined with silver doping. This method improves transistor performance and opens pathways toward applications in optoelectronics and neuromorphic computing [115].

Nikolaos et al. found that infrared (IR) sensors have widespread utility in detecting IR radiation, including industrial monitoring, environmental sensing, and security systems [57]. Leonardo et al. similarly observed that IR sensors are extensively employed for IR detection in fields such as medical diagnostics, industrial thermal imaging, and aerospace surveillance. Yet third-generation IR detector technology continues to encounter challenges. IR radiation is categorized into different transmission windows, and IR detectors find varied uses. While current high-performance IR imaging is largely based on certain epitaxially grown architectures, nanostructures and nanomaterials hold promise for improving detector characteristics. This manuscript reviews the types, materials, and applications of IR sensors and offers a summary and outlook [116]. Lu et al. noted that ultra-wide-bandgap Ga_2_O_3_ is highly promising for applications such as solar-blind photonics, owing to its outstanding optical and electrical properties in the deep-ultraviolet range; however, band alignments among its polymorphs have often been overlooked. In this work, a β-Ga_2_O_3_/κ-Ga_2_O_3_ phase heterojunction with a well-defined interface and type-II band alignment was experimentally demonstrated. The resulting self-powered deep-ultraviolet photodetector exhibits enhanced responsivity and faster response times. These findings provide valuable insights for Ga_2_O_3_ heterojunction interface studies and device applications [117]. Tanmoy et al. investigated the effect of molecular engineering on the optoelectronic properties of antimony corroles featuring two distinct β-substituents and two different antimony oxidation states. Introducing a strong electron-withdrawing SCN group onto the bipyrrole unit of the corrole was found to increase the molecular dipole moment [118].

**Figure 17:**
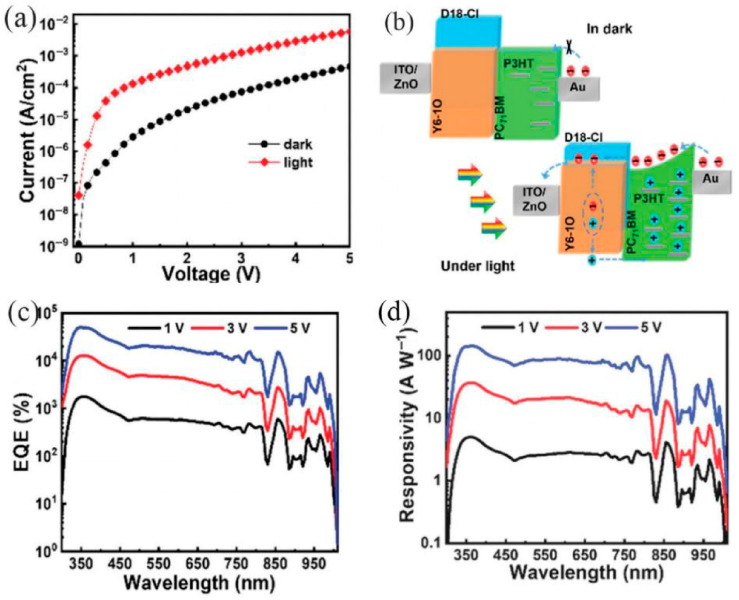
The double-layered OPMPDs (**a**) J–V curves. (**b**) Schematic diagram of the working mechanism. (**c**) EQE spectra under different applied voltages. (**d**) Responsivity spectra under different applied voltages [122]. Copyright 2021, Royal Society of Chemistry.

Efficient carrier transport is important for the performance of high-quality optoelectronic devices. This research employs an in situ fluorination method using benzoyl fluoride to mitigate bulk trap states in metal chalcogenide quantum dots, thereby enhancing charge carrier mobility. This method has been significantly improved and shows considerable potential for advancing the practical use of quantum dot-based technologies [99]. Posak et al. reported a novel MoS_2_/Co_3_O_4_ heterojunction based on bioplastic semiconductors, revealing an energy-band mechanism influenced by carrier concentration and demonstrating that the optimized device exhibits promising photodetector performance, advancing the use of bioplastics in electronics [69]. Transient electronics, which feature degradable devices that disappear after use, have attracted significant attention due to concerns about electronic waste. However, device degradability alone is insufficient for environmental safety; the evaluation of degradation by-products is critical. A study on two device types reveals that released by-products can be toxic or complex, highlighting the need for careful material selection and reassessment in transient electronics to mitigate end-of-life environmental impacts [119]. Larson et al. emphasized that indium phosphide nanowires are vital components for high-speed electronics and optoelectronic systems. Yet, conventional synthesis methods suffer from drawbacks, including high reaction temperatures and the toxicity of precursors. They developed a new solution-liquid-solid growth method at 180 °C using indium tris(trifluoroacetate) and tris(diethylamino)phosphine to synthesize thin zinc blende InP nanowires. By adjusting the indium precursor, this method enables morphological regulation and the growth of bulk InP nanowires [120]. Nguyen and co-workers explored the structural, electronic, and performance properties of photodetectors based on α-Ga_2_O_3_ with an MSM structure. By adopting first-principles calculations based on the GGA+U framework, they discovered that Al(111) and Ni(111) electrodes, when interfaced with α-Ga_2_O_3_(0001), undergo tensile strain and compressive strain, respectively [121]. A research team led by Liu fabricated broadband UV-Vis-NIR organic photomultiplication photodetectors (OPMPDs) with a precisely tailored device configuration. Controlled by the distribution of trapped holes, the devices exhibit a broad spectral response, high EQE (external quantum efficiency), outstanding specific detectivity, and excellent linear dynamic range, offering a viable approach to realizing high-performance broadband OPMPDs. The current density–voltage (J–V) properties of the bilayer OPMPDs were tested in the dark and under white light irradiation with an intensity of 2 Mw cm^−2^, as depicted in Figure 17a. Trapped holes within the P3HT layer adjacent to the Au electrode induce interfacial band bending, enabling adequate electron injection from the Au electrode (Figure 17b). As shown in Figure 17c, the EQE spectra of the bilayer OPMPDs measured under various forward bias voltages show a broad spectral response spanning 300 to 1000 nm, with EQE values surpassing 100%. The responsivity © spectra of the bilayer OPMPDs obtained via calculation are presented in Figure 17d.

**Figure 18:**
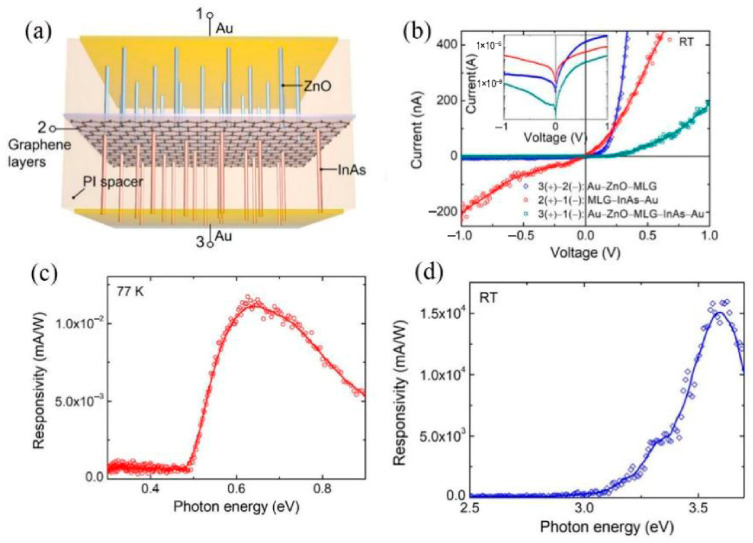
Photodetector device. (**a**) Schematic diagram of the device structure, and (**b**) current–voltage characteristic curves measured between three electrodes at room temperature (RT). Inset figure shows the semi-log plot. (**c**) Spectral response of the gold (Au)-InAs nanorod-graphene layers measured at 77 K using Fourier transform-infrared (FT-IR) spectroscopy. (**d**) Spectral response of the Au-ZnO nanorod-graphene layers measured at RT [124]. Copyright 2021, Springer Nature.

Shuang et al. noted that oxide materials with non-centrosymmetric structures exhibit a bulk photovoltaic effect (BPVE); however, photovoltaic cells based on these materials typically suffer from relatively low energy-conversion efficiency. In this work, a significant breakthrough is reported in enhancing BPVE in 3R-MoS_2_ by employing an edge-contact (EC) geometry with a bismuth semimetal electrode. This configuration induces strain and substantially boosts the BPVE-driven photocurrent. A 3R-MoS_2_/WSe_2_ heterojunction was also designed to demonstrate the coupling of BPVE with the conventional photovoltaic effect, highlighting its potential for applications in photodetectors and photovoltaic devices [123]. As reported by Tchoe et al., a multilayer graphene film was used as a suspended substrate to achieve the monolithic integration of InAs nanorods and ZnO nanotubes, enabling the fabrication of dual-wavelength photodetectors with a hybrid material architecture. The device structure is schematically shown in Figure 18a. At the tips of InAs nanorods and ZnO nanotubes, Au electrodes serve as Schottky contacts, while silver paste offers ohmic contact to the CVD-grown graphene layers. Figure 18b displays the current-voltage (I–V) characteristics measured among the three electrodes. The spectral response of the Au-InAs-MLG device (InAs nanorod side) produces a photocurrent when photon energies are above 0.5 eV, with peak responsivity emerging near 0.6 eV (Figure 18c), which aligns with previously published results. Simultaneously, the Au-ZnO-MLG Schottky photodiode (ZnO nanotube side) shows a photocurrent onset at photon energies higher than 3.1 eV and a responsivity peak at about 3.6 eV in its spectral response (Figure 18d).

Photodetectors underpin modern optoelectronics, but conventional silicon devices are hitting fundamental limits in flexible imaging, ultralow-light sensing and broadband communication. While silicon dominates the industry through mature CMOS compatibility, emerging materials offer superior performance but suffer from unaddressed stability, scalability and toxicity issues. Bridging the lab-to-fab gap remains the field’s defining challenge.

## 3. Conclusions and Outlook

In this work, we systematically reviewed and investigated several typical categories of photoelectric functional systems and materials. These include self-powered devices; UV, visible-light, near-infrared, and solar-blind photodetectors; core structural designs, such as P–N junctions; emerging materials, such as perovskite, graphene, and hybrid graphene/PbS quantum dots; composite systems, such as hybrid silicon–polymer structures; broadband-responsive devices; and other specialized systems. The findings indicate that these categories have both similarities and unique features. Each category has a wide range of applications. Self-powered photodetectors are especially suitable for wearable health monitoring in scenarios that require portability, sustainability and miniaturization. This is one of the primary application directions. Narrowband-responsive devices (UV, visible-light, near-infrared, and solar-blind) meet the demands of targeted spectral detection in specific fields. The efficient conversion of transmitted optical signals into electrical signals makes these devices core components of modern communication networks and highly compatible with the working principle of P–N photodetectors under reverse bias conditions. The depletion layer (space charge region) of the device enables efficient separation of photogenerated carriers, thereby producing a photogenerated current. Hybrid systems (e.g., hybrid silicon-polymer and hybrid graphene/PbS quantum dots) integrate the advantages of different materials to overcome the limitations of single-material systems. Broadband devices achieve full-spectrum detection, expanding application boundaries; other specialized systems further enrich the diversity of photoelectric detection technologies. However, many challenges exist. Some photodetectors are cumbersome and not eco-friendly. The integration of smart features and functional performance is also being pursued.

For future development, we propose the following directions: First, it is crucial to optimize the fabrication processes of high-performance materials. This would enable development and environmental care to go hand in hand. Second, hybrid photodetectors should be further explored to enhance sensitivity and broaden their application range. Third, practical application scenarios should be expanded. Self-powered technology can be integrated with narrowband and broadband detection systems to develop portable, low-power optoelectronic devices for wearable electronics and field sensing. Solar-blind detectors should be deployed in aerospace, flame detection, and other specialized fields by improving their resistance to interference.

Fourth, research on emerging and specialized systems should be strengthened. New functional materials and response mechanisms should be investigated to overcome existing technical barriers in photodetection. We believe that, with ongoing advances in materials science and device design, these photoelectric systems and materials will play increasingly vital roles in fields such as optoelectronic information, environmental monitoring, and biomedical sensing.

## Data Availability

Data sharing is not applicable to this article as no new data were created or analyzed.
